# Transcriptome Sequencing Reveals Novel Candidate Genes for *Cardinium hertigii*-Caused Cytoplasmic Incompatibility and Host-Cell Interaction

**DOI:** 10.1128/mSystems.00141-17

**Published:** 2017-11-21

**Authors:** Evelyne Mann, Corinne M. Stouthamer, Suzanne E. Kelly, Monika Dzieciol, Martha S. Hunter, Stephan Schmitz-Esser

**Affiliations:** a Institute for Milk Hygiene, Milk Technology and Food Science, University of Veterinary Medicine, Vienna, Austria; b Department of Entomology, University of Arizona, Tucson, Arizona, USA; University of California, Riverside

**Keywords:** *Bacteroidetes*, *Cardinium*, cytoplasmic incompatibility, host-microbe interaction, RNA sequencing, endosymbionts, gene expression

## Abstract

Cytoplasmic incompatibility (CI) is an intriguing, widespread, symbiont-induced reproductive failure that decreases offspring production of arthropods through crossing incompatibility of infected males with uninfected females or with females infected with a distinct symbiont genotype. For years, the molecular mechanism of CI remained unknown. Recent genomic, proteomic, biochemical, and cell biological studies have contributed to understanding of CI in the alphaproteobacterium *Wolbachia* and implicate genes associated with the WO prophage. Besides a recently discovered additional lineage of alphaproteobacterial symbionts only moderately related to *Wolbachia*, *Cardinium* (*Bacteroidetes*) is the only other symbiont known to cause CI, and genomic evidence suggests that it has very little homology with *Wolbachia* and evolved this phenotype independently. Here, we present the first transcriptomic study of the CI *Cardinium* strain *c*Eper1, in its natural host, *Encarsia suzannae*, to detect important CI candidates and genes involved in the insect-*Cardinium* symbiosis. Highly expressed transcripts included genes involved in manipulating ubiquitination, apoptosis, and host DNA. Female-biased genes encoding ribosomal proteins suggest an increase in general translational activity of *Cardinium* in female wasps. The results confirm previous genomic analyses that indicated that *Wolbachia* and *Cardinium* utilize different genes to induce CI, and transcriptome patterns further highlight expression of some common pathways that these bacteria use to interact with the host and potentially cause this enigmatic and fundamental manipulation of host reproduction.

**IMPORTANCE** The majority of insects carry maternally inherited intracellular bacteria that are important in their hosts’ biology, ecology, and evolution. Some of these bacterial symbionts cause a reproductive failure known as cytoplasmic incompatibility (CI). In CI, the mating of symbiont-infected males and uninfected females produces few or no daughters. The CI symbiont then spreads and can have a significant impact on the insect host population. *Cardinium*, a bacterial endosymbiont of the parasitoid wasp *Encarsia* in the *Bacteroidetes*, is the only bacterial lineage known to cause CI outside the *Alphaproteobacteria*, where *Wolbachia* and another recently discovered CI symbiont reside. Here, we sought insight into the gene expression of a CI-inducing *Cardinium* strain in its natural host, *Encarsia suzannae*. Our study provides the first insights into the *Cardinium* transcriptome and provides support for the hypothesis that *Wolbachia* and *Cardinium* target similar host pathways with distinct and largely unrelated sets of genes.

## Abstract (precis)

The majority of insects carry maternally inherited intracellular bacteria that are important in their hosts’ biology, ecology, and evolution. Some of these bacterial symbionts cause a reproductive failure known as cytoplasmic incompatibility (CI). In CI, the mating of symbiont-infected males and uninfected females produces few or no daughters. The CI symbiont then spreads and can have a significant impact on the insect host population. *Cardinium*, a bacterial endosymbiont of the parasitoid wasp *Encarsia* in the *Bacteroidetes*, is the only bacterial lineage known to cause CI outside the *Alphaproteobacteria*, where *Wolbachia* and another recently discovered CI symbiont reside. Here, we sought insight into the gene expression of a CI-inducing *Cardinium* strain in its natural host, *Encarsia suzannae*. Our study provides the first insights into the *Cardinium* transcriptome and provides support for the hypothesis that *Wolbachia* and *Cardinium* target similar host pathways with distinct and largely unrelated sets of genes.

## INTRODUCTION

Terrestrial arthropods are commonly associated with one or more intracellular, maternally transmitted bacterial symbionts that may profoundly influence their ecology and evolution (1, 2). Strictly maternally inherited symbionts spread in host populations by enhancing the daughter-producing capacity of female hosts relative to uninfected individuals (3). Obligate or “primary” symbionts are generally nutritional mutualists, thus increasing offspring production generally, while facultative or “secondary” symbionts may also benefit their hosts directly (2, 4) or manipulate host reproduction in ways that promote the production or fitness of infected females (5, 6). One of these symbiont-driven host manipulations is cytoplasmic incompatibility (CI). At its simplest, CI symbionts in the male host modify sperm such that only eggs with the same symbiont can “rescue” them, and the embryo develops normally. Conversely, mating between infected males and uninfected females generally results in embryo lethality. By depressing the relative fitness of uninfected females, the CI phenotype leads to an increase in the production of symbiont-infected females in the population (7, 8). CI is caused by three symbiont lineages: *Cardinium hertigii*, in the *Bacteroidetes*, and *Wolbachia pipientis* and a recently discovered clade of symbionts of a coconut beetle, in the *Alphaproteobacteria* (9). CI-causing *Wolbachia* is more prevalent among arthropods (~40% compared to ~9% for *Cardinium* [10, 11]) and has received considerable attention, including analyses of multiple sequenced genomes (12–19) and some elegant cytogenetic studies (20, 21). However, the molecular genetic basis of CI has been unresolved for some time and is just recently beginning to be understood. A recent study of *Wolbachia* strain *w*Pip in mosquitoes suggests that two adjacent genes may be important: *cidB* (wPa_0283) and *cidA* (wPa_0282) (22). Similarly, two genes carried in the WO prophage eukaryotic association module from *w*Mel (*cifA* [WD0631] and *cifB* [WD0632]) have also been shown to be able to recapitulate the CI phenotype (23). Homologs of these genes were highly expressed in the ovaries of the parasitoid wasp *Nasonia vitripennis* by *w*VitA, another CI *Wolbachia* strain (23). While a deubiquitylating domain in *cidB* was hypothesized to be important for CI in one study (22), this domain was not found to be conserved among CI strains in another (23).

*Cardinium* CI was much more recently discovered than *Wolbachia* CI (24), and its absence in model arthropod systems (particularly *Drosophila* and mosquitoes) as well as the minute size of the arthropod hosts in which CI *Cardinium* has been documented (parasitoid wasps and mites [e.g., references 24 and 25]) has made its study challenging. The first *Cardinium* CI genome showed only four homologous genes possibly involved in host-cell interaction with CI *Wolbachia*; these included a putative patatin-like phospholipase, an uncharacterized membrane protein, putative RNA helicase, and a cold shock protein. In spite of this, the cytological appearance of embryo death in CI *Cardinium*-affected hosts is broadly similar to that caused by CI *Wolbachia* (26). Further, the functional overlap of some protein families such as ankyrin repeat proteins between these two lineages leaves open the possibility of conserved host targets and functional convergence of the CI mechanism (26, 27).

Here, we sought insight into the highly expressed and sex-specific differentially expressed (DE) genes of the CI-inducing *Cardinium* strain *c*Eper1 in its natural host, *Encarsia suzannae* (Hymenoptera: Aphelinidae), with a transcriptome sequencing (RNA-Seq) approach. The genome of *c*Eper1 (27) provided candidate CI genes to evaluate for expression and the opportunity to highlight novel transcripts and hypothetical proteins that may need greater study. Additionally, the recently published genome of an apparently asymptomatic (28) strain of *Cardinium* in *Bemisia tabaci* (*c*BtQ1) (29) allowed us to compare and further posit functions for these genes. Our work represents the first *Cardinium* expression profile. In general, very few transcriptomes of arthropod endosymbionts have been sequenced until very recently (30–32). This may be because of the high level of technical difficulty of recovering enough RNA from uncultivable bacteria within eukaryotic hosts, combined with costs that have only recently become affordable. The potential value of this approach, however, is illustrated by a recent study in which the transcriptome of a defensive *Spiroplasma* showed a spiroplasma-encoded toxin, highly expressed only when its *Drosophila* host was parasitized by nematodes (31). Also, a thorough stage- and sex-specific transcriptomic analysis of *Wolbachia* closely related to the CI symbiont *w*Mel in *Drosophila melanogaster* provided the first insights into sex-biased expression by this symbiont (30). Other *Wolbachia* transcriptome studies examined the role of the mutualist *Wolbachia* in the native host tissues of the filarial nematode system (32–34), documenting immune system avoidance and ATP biosynthesis (32).

## RESULTS

### The *Cardinium hertigii c*Eper1 transcriptome—general features.

The *Cardinium hertigii c*Eper1 genome contains 835 predicted protein coding sequences (CDSs), with 782 chromosomal and 53 plasmid (pCher) genes. In our strand-specific RNA sequencing experiment, 445 million reads were generated in total, with an average of 74,094,307 reads per sample. Approximately 1% of the reads mapped to the *Cardinium hertigii c*Eper1 genome (Table 1). More than 60% of the reads mapped were mRNA reads. The mean theoretical redundancy of coverage was 24.7× ± 4.7× and 32.4× ± 7.4× for chromosomal and plasmid genes, respectively. Additionally, the single perfect match coverage was 94.5% for chromosomal and 89.6% for plasmid genes (median values over all replicates). Fifteen potential novel transcripts, of which seven were putative antisense RNAs of annotated genes, were identified (see Table S1 in the supplemental material). There was no differential expression of the novel transcripts in males and females, and the putative functions of these transcripts are unknown.

10.1128/mSystems.00141-17.2 TABLE S1  Potential novel *c*Eper1 transcripts and putative antisense RNAs, calculated with ReadXplorer. The parameter “number of read starts” counts the number of reads and filters low-coverage regions with a number of reads below a certain threshold. The threshold used to detect novel transcripts was set to a 10-read minimum. “Coverage increase” indicates the coverage increase in percent from one position to the neighboring one (for the detection of novel transcripts, the threshold was set to a minimum of 50% increase from the start of the transcript to the position before). “Transcript stop” indicates the position at the chromosome where the putative transcript ends. Download TABLE S1, PDF file, 0.03 MB. Copyright © 2017 Mann et al. 2017 Mann et al. This content is distributed under the terms of the Creative Commons Attribution 4.0 International license.

**TABLE 1:** Transcriptome sequencing read statistics, read processing, mapping, and coverage

Data set statistic	*c*Eper1 in *E. suzannae* by sex and replicate					
	Female			Male		
	1st	2nd	3rd	1st	2nd	3rd
Total no. of reads	80,080,835	73,121,816	66,679,042	63,836,426	81,372,753	79,474,970
Trimmed reads, no. (%)	77,353,052 (96.59)	70,355,505 (96.22)	64,410,445 (96.60)	61,843,438 (96.88)	78,712,070 (96.73)	76,899,760 (96.76)
Trimmed reads mapped to *C. hertigii c*Eper1 chromosome (genome size, 0.89 Mb), no. (%)	725,968 (0.94)	834,050 (1.19)	693,560 (1.08)	534,524 (0.86)	728,725 (0.93)	651,504 (0.85)
mRNA reads mapped to chromosomal genes, no. (%)	434,696 (59.87)	577,310 (69.21)	432,106 (62.30)	297,994 (55.75)	474,008 (65.05)	411,779 (63.20)
Reads assigned to genes involved in host-cell interactions,^a^ no. (%)	10,353 (2.38)	13,943 (2.42)	9,728 (2.25)	8,406 (2.82)	13,506 (2.85)	12,816 (3.11)
Reads assigned to transporter genes, no. (%)	23,327 (5.37)	30,589 (5.30)	20,910 (4.84)	17,040 (5.72)	25,305 (5.34)	23,663 (5.75)
Reads assigned to transposases, no. (%)	23,102 (5.31)	31,299 (5.42)	18,775 (4.34)	18,508 (6.21)	30,759 (6.49)	29,227 (7.10)
Theoretical redundancy of coverage	24.5×	32.5×	24.4×	16.8×	26.7×	23.2×
Single perfect coverage (*C. hertigii c*Eper1 chromosome), %	94.32	95.87	86.32	92.48	95.39	94.65
Trimmed reads mapped to *C. hertigii* pCher plasmid (genome size, 0.06 Mb), no. (%)	52,626 (0.07)	61,874 (0.09)	31,325 (0.05)	40, 882 (0.07)	63,651 (0.08)	64,554 (0.08)
mRNA reads mapped to plasmid genes, no.	37,892	44,723	23,215	28,795	45,146	44,688
Theoretical redundancy of coverage	32.8×	38.7×	20.1×	24.9×	39.1×	38.7×
Single perfect coverage (*C. hertigii* pCher plasmid), %	88.69	91.38	73.19	86.51	91.15	90.49

a Including ankyrin repeats, TPRs, and ubiquitin system-interacting genes.

**FIG 1:**
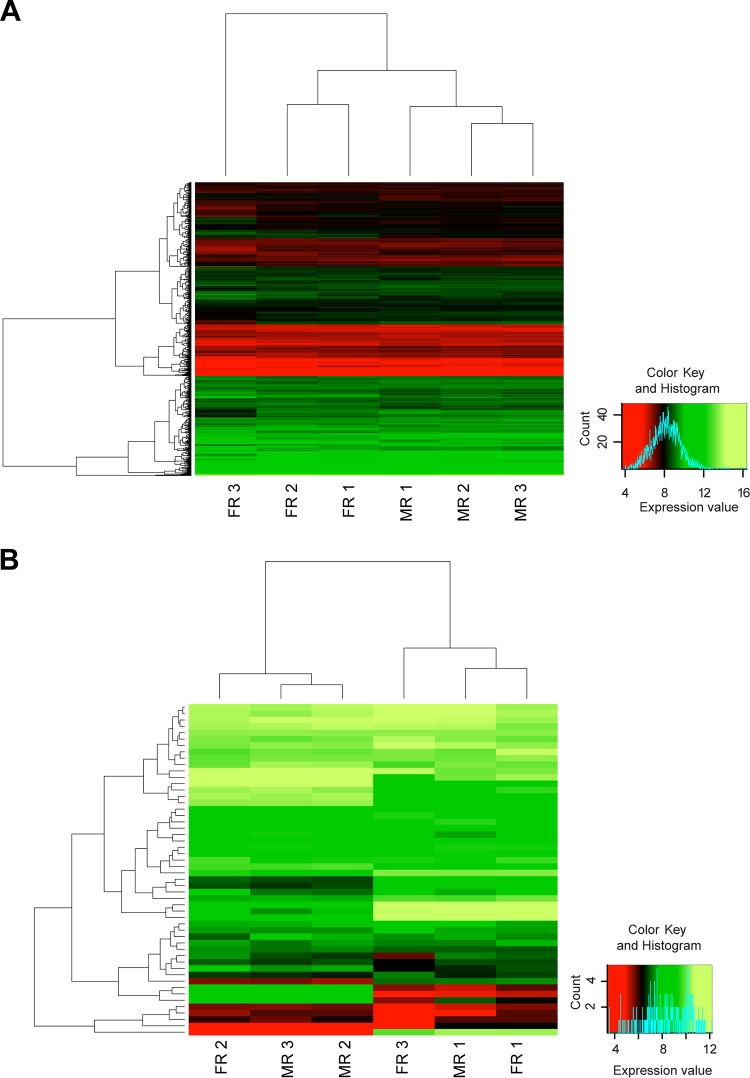
Gene expression of *c*Eper1 chromosomal (A) and plasmid (B) genes in female (FR) and male (MR) wasp pool replicates. tRNAs and rRNAs were excluded for all analysis. Expression values are given as transformed log values of normalized counts per gene (red, low expression; green, high expression). FR, *c*Eper1 genes in female wasp replicates; MR, *c*Eper1 genes in male wasp replicates.

Heat maps show a widely homogenous expression level of chromosomal genes among *c*Eper1 bacteria in male and female replicates (Fig. 1). Among the most highly expressed genes for *c*Eper1 across male and female replicates, many housekeeping genes (chaperones, genes involved in ribosomal machinery, and replication- or transcription-associated genes) were found (Table 2; Tables S2 to S4): for example, RNase P, 60-kDa chaperonin GroEL, and the DNA-directed RNA polymerase subunits beta and beta′. In female replicates, the DNA-directed RNA polymerase subunits, two elongation factors, one polyribonucleotide nucleotidyltransferase, and the signal recognition particle receptor FtsY were more highly expressed than in male replicates. In males, chaperone protein ClpB and one putative sodium-solute symporter appeared more highly expressed than in female replicates, but these differences were not statistically significant. The expression level of plasmid genes revealed some heterogeneity of expression that does not cleanly separate into male or female replicates, perhaps due to the absence of putative transcriptional regulators on the plasmids. In addition, several hypothetical proteins were found among the most highly expressed genes. Interestingly, we also found a high level of expression of transposases: 72 out of 129 transposases (55.8%) were expressed (Table S5), of which 18 were among the 100 most highly expressed genes. Although we cannot determine if these transposases are transpositionally active, the expressed transposases in *c*Eper1 could contribute to genomic recombination, as the *c*Eper1 and the *c*BtQ1 genomes show signs of substantial genomic rearrangements (29).

10.1128/mSystems.00141-17.3 TABLE S2  Classification of expression level of *c*Eper1 genes in female and male *Encarsia* wasps. Download TABLE S2, XLSX file, 0.1 MB. Copyright © 2017 Mann et al. 2017 Mann et al. This content is distributed under the terms of the Creative Commons Attribution 4.0 International license.

10.1128/mSystems.00141-17.4 TABLE S3  Thirty *c*Eper1 genes that were the most highly transcribed in male wasps are listed and sorted by fold change from their expression in female wasps. Genes meeting the DE criteria (*P* < 0.05 and multiple testing correction of FDR of <10%) are highlighted in bold. Download TABLE S3, PDF file, 0.03 MB. Copyright © 2017 Mann et al. 2017 Mann et al. This content is distributed under the terms of the Creative Commons Attribution 4.0 International license.

10.1128/mSystems.00141-17.5 TABLE S4  Thirty *c*Eper1 genes that were the most highly transcribed in female wasps are listed and sorted by the fold change from their expression in male wasps. Genes meeting the DE criteria (*P* < 0.05 and multiple testing correction of FDR of <10%) are highlighted in bold. Download TABLE S4, PDF file, 0.03 MB. Copyright © 2017 Mann et al. 2017 Mann et al. This content is distributed under the terms of the Creative Commons Attribution 4.0 International license.

10.1128/mSystems.00141-17.6 TABLE S5  Gene expression of transposases in the *Cardinium hertigii c*Eper1 genome. Download TABLE S5, PDF file, 0.1 MB. Copyright © 2017 Mann et al. 2017 Mann et al. This content is distributed under the terms of the Creative Commons Attribution 4.0 International license.

**TABLE 2:** The 20 most highly expressed *c*Eper1 genes in female and male wasp replicates^d^

Current GenBank locus tag^a^	Locus tag from reference 27^b^	Description	Mean *c*Eper1 rank in sex:	
			Males	Females
AL022_RS03910	—^c^	RNase P	1	1
AL022_RS03480	CAHE_0757	Hypothetical protein	2	2
AL022_RS01165	CAHE_0254	60-kDa chaperonin GroEL	3	3
AL022_RS00235	CAHE_0050	Hypothetical protein	4	4
AL022_RS03100	CAHE_0677	Putative DEAD box ATP-dependent RNA helicase	6	5
AL022_RS00080	CAHE_0016	Chaperone protein DnaK	5	6
AL022_RS01560	CAHE_0338	DNA-directed RNA polymerase subunit beta′	18	7
AL022_RS01520	CAHE_0330	Elongation factor Tu	12	8
AL022_RS03700	CAHE_0796	Putative sodium-solute symporter	7	9
AL022_RS01795	CAHE_0390	Hypothetical protein	8	10
AL022_RS02130	CAHE_0458	Putative phage tail sheath protein Afp4-like	10	11
AL022_RS01565	CAHE_0339	DNA-directed RNA polymerase subunit beta	34	12
AL022_RS00330	CAHE_0069	Elongation factor G	21	13
AL022_RS01865	CAHE_0406	Hypothetical protein	19	14
AL022_RS00520	CAHE_0112	Polyribonucleotide nucleotidyltransferase	20	15
AL022_RS02455	CAHE_0536	Chaperone protein ClpB	9	16
AL022_RS01915	CAHE_0417	Signal recognition particle receptor FtsY	27	17
AL022_RS00835	CAHE_0182	Putative chaperone protein Skp	17	18
AL022_RS01425	CAHE_0315	Chromosome partitioning protein ParA	16	19
AL022_RS02665	CAHE_0586	30S ribosomal protein S1	32	20
AL022_RS01815	CAHE_0394	Hypothetical protein	13	21
AL022_RS01610	CAHE_0352	Putative sodium-solute symporter	11	22
AL022_RS04180	CAHE_p0065	Putative transposase	15	34
AL022_RS04095	CAHE_p0043	Hypothetical protein	14	37

a GenBank accession numbers NC_018605.1 and NC_018606.1.

b GenBank accession numbers HE983995 and HE983996.

c —, not annotated in the work of Penz et al. (27).

d The ranking is based on mean normalized counts per gene of female and male wasp replicates.

### Expressed genes with eukaryotic domains: candidates for host-cell interaction and CI.

Many intracellular bacteria, including symbionts and pathogens, use proteins harboring eukaryotic domains such as ankyrin repeats or tetratricopeptide repeats (TPRs) to interfere with various host-cell functions (35, 36), including ubiquitination (37). *Cardinium c*Eper1 expressed many genes with eukaryotic domains that are candidates for host-cell interaction and/or the CI phenotype (Table 2). Fourteen out of 19 ankyrin repeat proteins identified in the genome were transcribed, with expression levels ranging from low to very high in *c*Eper1 (Table S6). Four moderately or highly expressed ankyrins on the *c*Eper1 chromosome showed high amino acid identity to homologs in *c*BtQ1 (CAHE_0095, 93%; CAHE_0435, 66%; CAHE_0680, 88%; CAHE_0834, 80%). In contrast, three highly expressed ankyrins located on the pCher plasmid were absent or nonfunctional in *c*BtQ1. While CAHE_p0007 and CAHE_p0014 have no homologs in *c*BtQ1, CAHE_p0026 has two pseudogene homologs on the *c*BtQ1 plasmid (see below). All three tetratricopeptide repeat (TPR) proteins present in the *c*Eper1 *Cardinium* genome (CAHE_0312, CAHE_0450, and CAHE_0452) were expressed moderately. Other transcripts with eukaryotic domains include CAHE_0028, a gene encoding a putative ubiquitin protease; CAHE_0010, with a WH2 motif and an N-terminal proline-rich domain commonly present in actin binding proteins; and CAHE_0286, a patatin-like phospholipase with high amino acid identity (64%) to homologs from WO prophages in *Wolbachia*. CAHE_0706 encodes a collagen-like protein that contains collagen triple helix repeats, and CAHE_0677 encodes a putative DEAD box ATP-dependent RNA helicase that was among the most highly expressed genes (Table 2). The DEAD box RNA helicase gene was predicted to be located within an operon together with the cold shock protein CAHE_0676, which is also among the most highly expressed genes (Table 2). CAHE_0676 and CAHE_0677 homologs are found in *Amoebophilus* and also in *Wolbachia* (27).

10.1128/mSystems.00141-17.7 TABLE S6  Transcription of *Cardinium hertigii c*Eper1 genes encoding proteins possibly involved in host-cell interactions. Download TABLE S6, PDF file, 0.04 MB. Copyright © 2017 Mann et al. 2017 Mann et al. This content is distributed under the terms of the Creative Commons Attribution 4.0 International license.

In addition to these previously identified candidate host-cell interaction genes, we also identified some novel effector candidates based on our RNA-Seq data. CAHE_0017 (moderately expressed) and CAHE_0267 (highly expressed) share 40% amino acid identity and are putative DNA-interacting proteins belonging to a previously described family of widely spread proteins harboring a MutS domain that affiliate with subfamily MutS8 (InterPro domains IPR007696, IPR027417, and IPR000432) (38, 39). Another highly expressed novel host-cell interaction candidate protein identified here is CAHE_0662, an integral membrane protein harboring an inhibitor of apoptosis-promoting Bax1 domain (InterPro domain IPR006214).

### Sex-specific transcription of genes.

**FIG 2:**
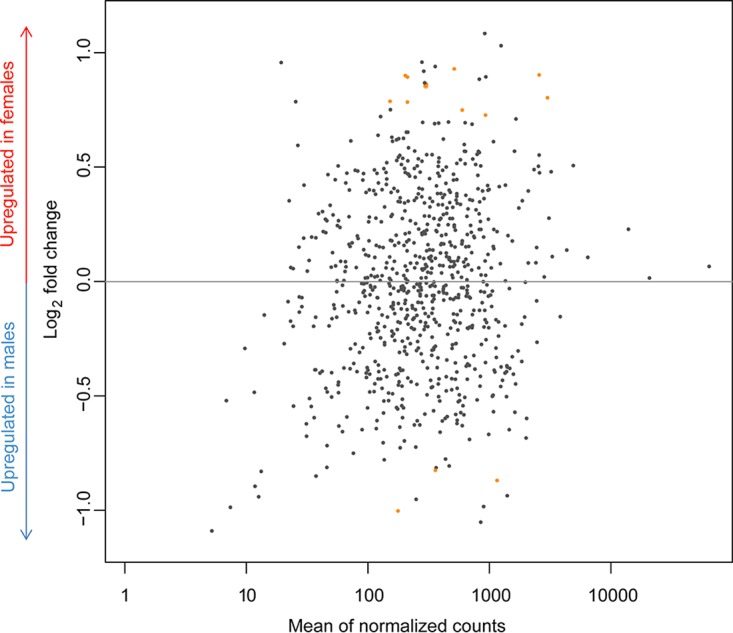
Normalized mean transcription values of *c*Eper1 chromosomal and plasmid genes are plotted against log_2_ fold change values for each gene. Differentially expressed (DE) genes are highlighted in orange.

With the RNA-Seq approach, 15 differentially expressed (DE) genes were found, of which 12 were upregulated in *c*Eper1 found within females and three in *c*Eper1 in males (Fig. 2; Table 3). DE genes were moderately expressed, except for the DNA-directed RNA polymerase subunits and CAHE_p0026, which were among the most highly expressed genes overall. In females, the DE genes consisted largely of genes involved in transcription and translation: five DE genes encoding ribosomal proteins were upregulated, indicating an increased general translational activity. In males, three DE genes were upregulated, including the previously mentioned CAHE_p0026, a putative RING domain ubiquitin ligase (InterPro domain IPR001841) located on the plasmid pCher, which also contains ankyrin repeats and was identified as a putative CI candidate gene previously (27). A second gene with higher expression in males was CAHE_p0027, a hypothetical protein with a homolog only in *c*BtQ1 (58% amino acid identity). Although CAHE_p0026 and CAHE_p0027 are both located on the pCher plasmid and adjacent to each other, these two genes are transcribed in opposite directions and are thus not part of an operon. The third gene upregulated in males is CAHE_0544; while the function of this gene is unknown, it contains a putative P-loop containing a nucleoside triphosphate hydrolase domain (InterPro domain IPR027417), and its only homolog is a truncated pseudogene in *c*BtQ1.

**TABLE 3:** List of *c*Eper1 DE genes determined with RNA sequencing and DE calling in DESeq^c^

Category and current GenBank locus tag^a^	Locus tag from reference 27^b^	Description	Mean expression value for *c*Eper1 in:		Log_2_ fold change	*P* value	FDR value
			Males	Females			
Genes upregulated in *c*Eper1 in females							
AL022_RS00605	CAHE_0131	30S ribosomal protein S5	353.56	673.17	0.93	<0.001	0.022
AL022_RS01565	CAHE_0339	DNA-directed RNA polymerase subunit beta′	1,783.38	3,335.95	0.90	<0.001	0.009
AL022_RS02585	CAHE_0565	Transcription elongation factor GreA	141.55	264.19	0.90	0.001	0.052
AL022_RS00600	CAHE_0130	50S ribosomal protein L30	147.84	274.74	0.89	<0.001	0.052
AL022_RS01110		Aspartate-tRNA ligase	214.87	390.65	0.86	<0.001	0.040
AL022_RS01125	CAHE_0242	Dipeptide transport system permease protein OppC	212.17	384.43	0.86	<0.001	0.040
AL022_RS02215	CAHE_0475	30S ribosomal protein S9	213.37	385.64	0.85	0.001	0.052
AL022_RS01560	CAHE_0338	DNA-directed RNA polymerase subunit beta—*rpoB*	2,191.78	3,822.27	0.80	<0.001	0.035
AL022_RS00610	CAHE_0132	50S ribosomal protein L18	111.66	192.77	0.79	0.001	0.079
AL022_RS03105	CAHE_0678	Hypothetical protein	155.67	268.09	0.78	0.001	0.079
AL022_RS00470	CAHE_0102	Membrane protein insertase YidC	446.65	750.46	0.75	0.001	0.052
AL022_RS01545	CAHE_0335	50S ribosomal protein L1	701.12	1,160.67	0.73	0.001	0.057
Genes upregulated in *c*Eper1 in males							
AL022_RS02490	CAHE_0544	Hypothetical protein	459.68	259.51	−0.82	0.002	0.095
AL022_RS04030	CAHE_p0026	RING domain-containing protein, ankyrin repeats	1,491.23	816.33	−0.87	0.001	0.062
AL022_RS04035	CAHE_p0027	Hypothetical protein	235.71	117.67	−1.00	0.001	0.052

a GenBank accession numbers NC_018605.1 and NC_018606.1.

b GenBank accession numbers HE983995 and HE983996.

c Mean expression values are normalized counts per gene of female and male wasp replicates.

**FIG 3:**
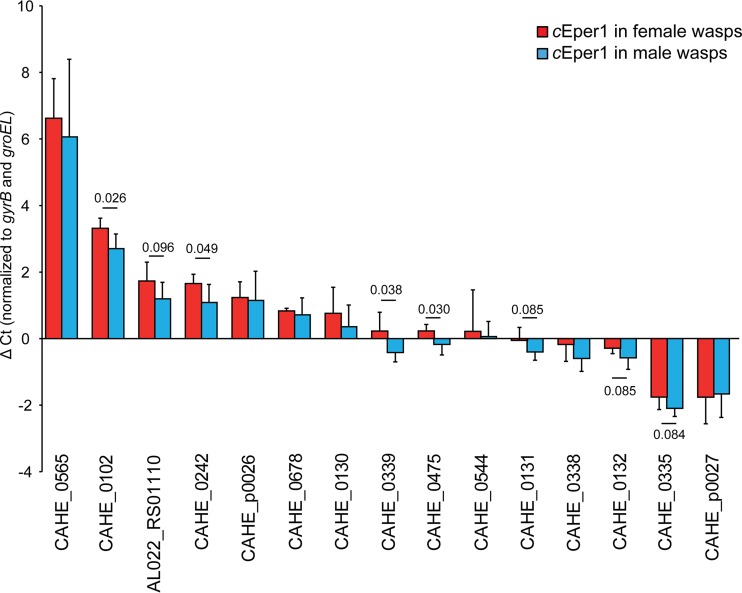
RT-qPCR confirmation of *c*Eper1 DE genes. RT-qPCR was done with three replicates per sex which were also used for the RNA-Seq experiment and with two independent replicates per sex. The expression level of DE genes was normalized to that of the two housekeeping genes *gyrB* and *groEL*. *P* values were listed for genes which were confirmed to be differentially expressed (significantly or by trend).

To confirm the accuracy of expression profiles obtained from the transcriptome sequencing experiment, independent validation of DE genes was performed with reverse transcriptase quantitative PCR (RT-qPCR). First, the same replicates used for RNA-Seq (except for one female replicate where no RNA was left after sequencing) were examined. A strong correlation between transcriptional differences (fold changes between male and female replicates) measured by RNA-Seq and RT-qPCR was found (regression *P* < 0.001; correlation coefficient *r* = 0.98; Fig. S1), providing strong evidence for the quantitative accuracy of the RNA data set. This analysis revealed the same trends in regulation of all 15 DE genes tested relative to the RNA-Seq experiment. Second, DE genes of two independent replicates of males and females were examined with RT-qPCR to test if these expression patterns are constant and reproducible across samples. Sex-enriched expression was confirmed for 8 out of 15 DE genes (Fig. 3). The three male-specific genes were not confirmed in the independent replicates by RT-qPCR, indicating a diversified expression pattern among samples.

10.1128/mSystems.00141-17.1 FIG S1  (A) Fold change differences of sex-specific expression patterns detected with RNA-Seq and RT-qPCRs are compared. (B) The correlation of fold change differences between the two methods used is shown. In panels A and B, only replicates that were used for both methods (three male and three female replicates) were included in the calculation. Download FIG S1, TIF file, 2.7 MB. Copyright © 2017 Mann et al. 2017 Mann et al. This content is distributed under the terms of the Creative Commons Attribution 4.0 International license.

### *Cardinium* metabolism, transporters, and secretion system.

*Cardinium* has highly reduced metabolic capabilities and encodes only two complete biosynthetic pathways (27, 29): the B-vitamin biotin biosynthesis and the lipoate pathway, all genes of which were expressed. The expression level for most of these genes was moderate; only the biotin synthase BioB (CAHE_0559) was highly expressed. Perhaps to compensate for reduced metabolic capabilities, *c*Eper1 encodes 62 transport proteins, all expressed. Four moderately expressed putative nucleotide transport proteins were found in *Cardinium*: CAHE_0018, CAHE_0158, CAHE_0160, and CAHE_0789, all of which belong to the ATP:ADP antiporter family. *c*Eper1 also expresses a putative *S*-adenosylmethionine transporter (CAHE_0109, moderate expression), which shows 47% amino acid identity to the functionally characterized homolog from *Amoebophilus asiaticus* (40) and 93% amino acid identity to a homolog in *c*BtQ1 (29). The transport system Opp A-F (CAHE_0240 to _0242, _0244, and _0245) and the C_4_-dicarboxylate transporter DcuAB (CAHE_0645 and _0647) were moderately expressed, indicating a functional import system for oligopeptides, amino acids, and dicarboxylates. *Cardinium c*Eper1 also encodes 12 putative sodium-solute symporters, all expressed. Two of them (CAHE_0796 and CAHE_0352) were among the most highly expressed genes overall. Last, we found transcripts of all 15 genes identified previously as a novel, putative phage-derived protein secretion system (27, 41, 42) (Table S7). Some of these antifeeding prophage (AFP) genes were among the highly expressed genes, including the putative phage tail sheath protein (CAHE_0458) and the AFP11-like phage baseplate protein (CAHE_0037).

10.1128/mSystems.00141-17.8 TABLE S7  Transcription of *Cardinium hertigii c*Eper1 genes encoding proteins of the putative antifeeding prophage-like secretion system. Download TABLE S7, PDF file, 0.03 MB. Copyright © 2017 Mann et al. 2017 Mann et al. This content is distributed under the terms of the Creative Commons Attribution 4.0 International license.

## DISCUSSION

### Candidate genes for host interaction and CI.

The *Cardinium* strain *c*Eper1 expresses a number of genes with eukaryotic domains, signal peptides directing secretion from the bacterial cell, or other genes that are likely involved with host cells, some of them potentially CI candidates, some likely more generally involved in symbiosis. While genomic data show independent evolution of CI in *Cardinium* and *Wolbachia* (12–16, 27, 43), the evidence to date suggests convergence in the CI phenotype (26), suggesting that the molecular targets may be similar. Further, some of the patterns of gene expression suggest possible convergence of function between the two lineages and between *Cardinium* and other symbionts.

### Candidates for manipulation of host ubiquitination system.

Recent research suggesting that CI *Wolbachia* may target the host ubiquitination system, a key regulatory process in eukaryotes (22, 44), makes the presence of highly expressed ubiquitin ligase and protease genes in *Cardinium* especially intriguing. Caution is warranted, however, since it is not clear that, in *Wolbachia* (*w*Pip) CI, the rescue is achieved via restoration of ubiquitination, and the region of homology of one of the CI candidate genes across *Wolbachia* CI strains does not include the ubiquitin protease domain (23). Further, *c*Eper1 does not harbor homologs of *Wolbachia cidA* and *cidB* but does express CAHE_p0026, a putative RING domain ubiquitin ligase with ankyrin repeats, located on the plasmid pCher. Many bacterial effector proteins interfering with the host ubiquitin system mimic host structures or motifs, and RING domain proteins have been shown to mimic RING-type E3 ubiquitin ligases (37, 45). The only significant homologs of CAHE_p0026 were found on the *Cardinium c*BtQ1 plasmid pcBtQ1 (37% and 48% amino acid identity), but both *c*BtQ1 genes are disrupted by a transposase and are therefore nonfunctional pseudogenes. Interestingly, the *Cardinium* strain *c*BtQ1 does not appear to cause CI or another known reproductive manipulation of its host and is considered asymptomatic (28, 29). In addition to the RING domain ubiquitin ligase, CAHE_0028, a gene encoding a putative ubiquitin protease, is also highly expressed. Ubiquitin proteases are present in a few symbiotic and pathogenic bacteria, e.g., in *Arsenophonus nasoniae*, a male-killer bacterium of *Nasonia vitripennis*, and in the pathogens *Chlamydia trachomatis* and *Salmonella enterica* serovar Typhimurium (37, 46, 47). The ubiquitin protease of *Cardinium* is conserved in *c*BtQ1 as well (27, 29) and could work in concert with the ligase in contributing to the CI phenotype or in host manipulation generally.

### Candidates for manipulation of host DNA.

The phenotype of both dying CI *Wolbachia* and CI *Cardinium-*influenced embryos includes improper condensation of host chromosomes and disrupted cell cycle timing of mitotic divisions (20, 26). Several expressed *c*Eper1 genes are likely to interact with host chromatin. CAHE_0677 encodes a putative DEAD box ATP-dependent RNA helicase and was among the most highly expressed genes. Eukaryotic DEAD box RNA helicases promote mitotic chromosome segregation together with the RNA interference pathway (48). *Cardinium c*BtQ1, *Wolbachia*, and the closest relative to *Cardinium*, the amoeba symbiont *Amoebophilus asiaticus*, also harbor a DEAD box ATP-dependent RNA helicase gene that is highly similar to *c*Eper1 (98%, 54%, and 52% amino acid identity, respectively). The helicase gene was predicted to be located within an operon together with the cold shock protein CAHE_0676, which is also a highly expressed gene. In addition, two novel putative DNA-interacting candidates were identified from our RNA-Seq data: CAHE_0017 (moderately expressed) and CAHE_0267 (highly expressed) harbor a MutS domain. MutS homologs are predicted to be involved in DNA mismatch repair or recombination and to be critical for replication fidelity and genome stability in both prokaryotes and eukaryotes (38, 39). Defects in the mismatch repair system can lead to meiotic defects. Somewhat surprisingly for bacterial DNA-interacting proteins, both *Cardinium* proteins harbor a predicted signal peptide, indicating that they are secreted from the bacterial cell. Interestingly, *Cardinium c*BtQ1 and *A. asiaticus* have also two genes homologous (with 36% and 92% amino acid identity, respectively) to CAHE_0017 and CAHE_0267. The homologs in *c*BtQ1 also contain a predicted signal peptide, whereas the *A. asiaticus* homologs do not.

### Other candidates for host manipulation.

Another novel host-cell interaction candidate protein identified here is CAHE_0662, an integral membrane protein harboring an inhibitor of the apoptosis-promoting Bax1 domain (InterPro domain IPR006214), also among the most highly expressed genes (Table 2). Eukaryotic homologs are also known as Golgi antiapoptotic protein (GAAP) or as transmembrane Bax inhibitor motif (TMBIM) family proteins. These homologs localize predominantly to the Golgi apparatus and have been shown to inhibit apoptosis by releasing Ca^2+^ stored in the endoplasmic reticulum (ER) and the Golgi apparatus (49). CAHE_0662 has homologs in *Cardinium c*BtQ1 (85% amino acid identity), and more distant homologs (with approximately 40% amino acid identity) can be found in *Orientia*, *Rickettsia*, and plant-associated *Alphaproteobacteria* (49). More generally, nonhomologous putative apoptosis-inhibiting genes have recently been found in the WO prophage eukaryotic association modules in *Wolbachia* (50), and apoptosis inhibition by *Wolbachia* has also been implicated in preserving normal ovarian development in a parasitoid wasp system in which the host is dependent on the symbiont (51). The *Cardinium* GAAP-like protein shows 28% amino acid identity to the human homologs and harbors a number of amino acid residues which have been shown to be essential for function of GAAPs (49, 52). We thus hypothesize that CAHE_0662 is a protein involved in host-cell interaction, perhaps by inhibiting apoptosis of infected host cells.

Two other host-cell interaction candidate genes identified in the *c*Eper1 genome were moderately expressed. CAHE_0010 contains a WH2 motif and a proline-rich domain at the N terminus, often part of actin binding proteins. A homolog of CAHE_0010 was found in *c*BtQ1 (46% amino acid identity). CAHE_0286, a patatin-like phospholipase, has high amino acid identity (64%) to homologs from WO prophages in *Wolbachia*. WO prophages appear to play an important role in reproductive manipulation in *Wolbachia*, as highly sex-specific expression patterns have been detected for WO prophage genes (43, 53, 54). Several proteins associated with WO prophage regions are reproductive manipulation candidates, and there is a concentration of genes with eukaryotic domains in the eukaryotic association module of the WO phage (50). More recently, the involvement of *cifA* and *cifB*, two genes in the eukaryotic association module of WO prophage, in CI has been described by LePage and coworkers (23), providing strong evidence for an involvement of WO prophages in *Wolbachia* CI. Similarly to the recently described *cidA* and *cidB*, *c*Eper1 does not harbor homologs of *cifA* and *cifB*.

The CI *Cardinium* transcriptome also highlighted which of hundreds of hypothetical proteins could be important in metabolism or host manipulation. Another putative host-cell interaction protein might be CAHE_0757, which is the second most highly expressed gene in both female and male *c*Eper1. CAHE_0757 has only one hit in GenBank, in *c*BtQ1; however, this homolog shows only 28% amino acid identity to CAHE_0757. Interestingly, CAHE_0757 is predicted to contain a signal peptide as well.

### Ankyrin repeat domain and tetratricopeptide repeat domain proteins.

Ankyrin proteins are significantly enriched in many intracellular host-associated bacteria compared to free-living bacteria and are likely part of an effective interaction system between bacterial and host proteins. Interestingly, CI-inducing *Cardinium hertigii*, *A. asiaticus*, and *Wolbachia* strains are particularly enriched in ankyrin proteins, while they are absent or rare in other *Bacteroidetes* and mutualistic *Wolbachia* strains (12, 13, 27, 55, 56). Ankyrin proteins mediate protein-protein interactions in eukaryotes (57), and some intracellular bacteria secrete ankyrin proteins to manipulate host-cell functions (58–62). Ankyrin repeat proteins have long been considered potential CI effectors in *Wolbachia* spp., and some studies showed sex-specific gene expression of ANK genes (30, 43, 53, 63–65), but their direct role in *Wolbachia* CI is unclear (65, 66), and targeted studies have generally failed to confirm a direct role of these abundant proteins in *Wolbachia* CI (65, 66). The ankyrin repeat proteins found in both *Wolbachia* and *Cardinium* genomes share no similarity except for the shared ankyrin repeat motif.

Three tetratricopeptide repeat (TPR) genes were moderately expressed (CAHE_0312, CAHE_0450, and CAHE_0452) and may also be important in host manipulation. TPRs often have central roles in vital cell processes in eukaryotes, and they may be directly related to the virulence of bacterial pathogens (35). Proteins containing TPRs can regulate defined cell cycle transitions, for example, the anaphase-promoting complex in eukaryotes (67), and were also found in high numbers in *A. asiaticus* and in *Chlamydiae*, *Orientia*, and nematode *Wolbachia* genomes (55, 56, 68, 69). Like the ankyrins, the TPR *Cardinium* genes expressed show no homology to *Wolbachia* other than the TPR domain.

### *Cardinium* metabolism, transporters, and secretion system.

Like many other intracellular symbionts, the *c*Eper1 *Cardinium* showed a highly reduced metabolic capability and dependence on a large assortment of transporters. Complete pathways for lipoate and biotin biosynthesis were expressed at moderate levels. Lipoate is a highly conserved sulfur-containing cofactor that is essential for the function of key enzymatic processes. The acquisition and use of lipoate are also associated with bacterial virulence and pathogenesis (70). The expression of a complete biotin biosynthesis pathway is more surprising. The pathway is incomplete in the whitefly *Cardinium* strain *c*BtQ1, suggesting that it is not necessary for symbiont metabolism. It also seems unlikely to be required by the host. Biotin is typically ingested by insects (71), and although it may be supplied by symbionts to blood feeders whose diet customarily lacks B vitamins (72), parasitoids like *Encarsia* wasps that consume whole insects are unlikely to have dietary imbalances. Further, *Encarsia suzannae* insects cured of their *Cardinium* symbionts are able to survive and reproduce normally (e.g., references 73 and 74. The role of this vitamin in the wasp-*Cardinium* interaction is therefore unclear.

We found moderate to high transcription of all 15 genes in the unusual putative phage-derived protein secretion system identified in the *Cardinium* genome (27, 41). The secretion system is related to the antifeeding prophage (AFP) from *Serratia entomophila* and to other phage-derived secretion systems (42, 75, 76). While the whitefly *Cardinium* strain *c*BtQ1 (29) has a putative type I secretion system, this is absent from *c*Eper1, and no other known protein secretion system was documented (27). In the current study, we found that some AFP genes were among the most highly expressed genes, suggesting a substantial functional role of the AFP apparatus in communication with the host cell. While CI candidate genes in *Wolbachia* might be secreted by a type IV secretion system, in *Cardinium*, CI candidate genes may be translocated into the host by the AFP-like protein secretion system. Recent studies have shown that AFP-like genes are not phylum specific but widespread among various bacterial and archaeal lineages (76) and that an AFP homolog of the symbiont *Pseudoalteromonas luteoviolacea* is responsible for induction of metamorphosis of the tubeworm *Hydroides elegans* (75). A recent study showed that the *Amoebophilus asiaticus* AFP-like gene cluster represents a functional contractile secretion system (42); this novel secretion system may contribute structurally to the regular array of tubes visible in electron micrographs of *Cardinium* (42, 77–79).

### Conclusions.

Here, we provide the first insight into gene expression of the CI-causing *Cardinium* strain cEper1 in its natural host. This bacterium shows very little homology to CI *Wolbachia*, but *Cardinium* expression patterns suggest that the two symbionts may target at least some of the same host pathways. In *Cardinium c*Eper1, this includes a highly expressed RING domain ubiquitin ligase potentially targeting the same host pathway as genes that have been implicated in ubiquitin manipulation in CI *Wolbachia* (22). Other highly expressed *Cardinium c*Eper1 candidates that show functional similarity to *Wolbachia* genes include a DEAD box ATP-dependent helicase, an apoptosis-inhibiting gene (50), and ankyrin repeat domain protein genes (43, 63). To analyze the role of CI candidates in more detail in future experiments, it would be interesting to look for *Cardinium* proteins associated with infected male *E. suzannae* sperm (e.g., reference 44). To better understand the *Cardinium-*host interaction more generally, it would also be valuable to express *Cardinium* CI candidate proteins in a heterologous host and then use the recombinant proteins as “bait” to identify interacting host proteins. In addition, comparing the host gene expression data from infected and uninfected hosts, especially perhaps in male and female ovaries and testes, may also provide valuable complementary insights into the molecular basis of CI in this symbiotic system.

## MATERIALS AND METHODS

### *Encarsia suzannae* cultures.

*Encarsia suzannae* (previously known as *Encarsia pergandiella* [73]) is a parasitoid wasp (Hymenoptera: Aphelinidae) infected with the CI symbiont *Cardinium hertigii c*Eper1. Wasps were collected from their host whiteflies (*Bemisia tabaci*) in Weslaco, TX, in 2006 (80, 81). The wasps were cultured on whiteflies that were not infected with *Rickettsia*, on cowpea plants (*Vigna unguiculata*). Males of *E. suzannae* develop as hyperparasitoids and were cultivated by providing virgin adult females with late-instar larvae or early-stage pupae of the primary parasitoid *Eretmocerus eremicus*. Since female *E. suzannae* insects are primary parasitoids, whitefly nymphs were provided to mated, adult females for female wasp production. Therefore, males and females of *E. suzannae* were cultivated separately in 50-cm^3^ cages (27°C, ambient relative humidity).

Male and female wasps were cultivated in four cages per sex. All leaves bearing wasp pupae in one cage were placed in an emergence jar (81) and resulted in 350 to 500 1- to 3-day-old wasps of one sex. Since each wasp weighs approximately 18.68 μg (data not shown), the starting weight of each sample was approximately 6.54 to 9.34 mg, roughly equivalent to the weight of four to six *Drosophila melanogaster* females (82). At day 3, all wasps in each jar were collected into one tube and subsequently shock frozen at −80°C for 2 min.

### RNA extraction, HiSeq 2500 sequencing, and sequencing data analysis.

Total RNA was extracted from all eight single-sex pools. RNA was isolated immediately after wasps were shock frozen. For RNA isolation, the Trizol reagent (Invitrogen) was used, and contaminating genomic DNA was digested with the Turbo DNA-free kit (Ambion) according to the manufacturer’s instructions. After DNase treatment, RNA was dissolved in 15 µl double-distilled water with diethyl pyrocarbonate (ddH_2_O_DEPC_), and the complete digestion of DNA was confirmed by PCR with a 16S rRNA gene targeting general bacterial primer panel 27F-1492R (83). The integrity of the purified RNA was verified with an Agilent 2100 bioanalyzer (Agilent Technologies). RNA was stored at −80°C until use. Samples were subjected to standard Illumina library preparation using the NEBNext Ultra RNA library prep kit according to the manufacturer’s instructions. An RNA-Seq test run with different rRNA removal kits revealed that the best *c*Eper1 transcriptome coverage was achieved with the Ribo-Zero Magnetic Gold (epidemiology) kit (Epicentre Biotechnologies; data not shown), so this kit was used. Six double-stranded cDNA libraries, three for each sex, were single end sequenced (50 bp) using an Illumina HiSeq2500 machine at the Vienna Biocenter Core Facilities (VBCF) NGS unit (http://www.vbcf.ac.at). Sequences were quality filtered with mothur (84) using trimming parameters as follows: number of ambiguous bases allowed = 0, minimum length of reads = 30 bp, minimum average quality score allowed over a window of 10 bp = 25, maximum length of homopolymers = 8 bp. Quality-filtered reads were mapped to the *Cardinium hertigii* chromosome and plasmid, NCBI RefSeq NC_018605.1 and NC_018606.1 (27), respectively, in the Burrows-Wheeler aligner (85). Most genome annotations are based on our original automatic genome annotation of the *c*Eper1 genome using MicrScope/MaGe, which was then verified by manual searches of proteins against Swiss-Prot and UniProt, as well as searches against PFAM and SMART (see reference 27 for details). Read counts per predicted gene were calculated by ReadXplorer (86) and imported in DESeq Bioconductor using the R software environment (87). Gene counts were normalized to size factors of libraries and dispersion estimation. Normalized gene expression values are listed as normalized read counts per gene. An average normalized read count of >1,000 (top 10% of all genes) was considered highly expressed, and a read count of <60 (last 10% of all genes) was considered to show a low expression level. All genes with read counts between 60 and 1,000 were classified as moderately expressed. Differentially expressed (DE) genes between *c*Eper1 in the male and female pool replicates were determined by DESeq using a binomial distribution model (88). Genes were considered DE if *P* was <0.05 and if multiple testing correction of false discovery rate (FDR) was <10% (89). Putative novel transcripts were detected with the “Transcription Start Site Detection” option in ReadXplorer, considering the number of read starts at the position and the minimal coverage increase from one position to the next (86).

### Confirmation of DE genes with RT-qPCR.

For the RT-qPCRs, the first RNA from the same three replicates was used as for the RNA sequencing experiment, except for one female replicate where no RNA was left after sequencing. This replicate was replaced by the fourth biological replicate of RNA extracted from females. Second, four additional independent replicates, which were not used for RNA-Seq, were produced (two from females and two from males) to test if the gene expression patterns were reproducible among samples.

Transcription into cDNA was made with random hexamer primers (RevertAid H Minus First Strand cDNA synthesis kit; Thermo Scientific) and 5 µl RNA. RNA secondary structures were broken up at 65°C for 5 min, and cDNA synthesis was done at 45°C for 60 min after a preincubation at 25°C for 5 min. The reaction was terminated by heating at 70°C for 5 min, and cDNA was stored at −20°C.

Primers targeting DE genes and two housekeeping genes (*gyrB* and *groEL*) were designed using Primer 3 (version 0.4.0) (90) and Primer-BLAST (91). *In silico* specificity screens were done with BLAST. Annealing temperatures were optimized with genomic DNA isolated from *E. suzannae* infected with *c*Eper1 (Table S8).

10.1128/mSystems.00141-17.9 TABLE S8  Primer sequences, amplicon length, and annealing temperature of primers targeting *c*Eper1 DE genes and two housekeeping genes (*gyrB* and *groEL*). Annealing temperatures were optimized with genomic DNA isolated from a culture of *E. suzannae* infected with *Cardinium hertigii c*Eper1. Download TABLE S8, PDF file, 0.03 MB. Copyright © 2017 Mann et al. 2017 Mann et al. This content is distributed under the terms of the Creative Commons Attribution 4.0 International license.

RT-qPCR was performed according to the MIQE guidelines (92), listed in Table S9. Each qPCR mixture was pipetted in duplicate with Brilliant III SYBR Green qPCR low-ROX master mix, according to the manufacturer’s instructions (Agilent Technologies). All primers were used at a final concentration of 250 nM. All reactions were performed with an initial denaturation step at 95°C (3 min), followed by 40 cycles of 95°C for 5 s and annealing for 20 s with a fluorescence measurement at the last step of each cycle. A melting curve, ranging from 70°C to 90°C, with fluorescence measurements at 1°C intervals, was done after all real-time PCRs, to determine the specificity of the reaction. qPCRs were performed using a Stratagene Mx3000P real-time PCR system (Agilent Technologies).

10.1128/mSystems.00141-17.10 TABLE S9  MIQE guidelines for RT-qPCR assays. Download TABLE S9, PDF file, 0.1 MB. Copyright © 2017 Mann et al. 2017 Mann et al. This content is distributed under the terms of the Creative Commons Attribution 4.0 International license.

For inhibition testing and to evaluate the efficiencies of the DE and housekeeping gene PCR assays, standard curves were pipetted with purified PCR products of cDNA samples, which were adjusted to 1 ng/µl with a Qubit 2.0 fluorometer (Life Technologies). Negative-control and reverse transcriptase (RT)-minus controls (reverse transcription reaction without addition of reverse transcriptase) were used.

For the mRNA quantitation, 0.1 ng cDNA was used as the template in each qPCR. Data were analyzed using Mx300P MxPro software (Stratagene), and relative quantitation was performed with the comparative threshold cycle (*C*_*T*_) method. Values were normalized using two housekeeping genes (*gyrB* and *groEL*). Significant differences in bacterial gene expression between male and female *c*Eper1-positive pool replicates were determined with the Welch two-sample *t* test in R (version 3.2.0) with genes being considered DE if *P* was <0.05.

### Sequence analysis of highly expressed and differentially expressed genes.

Highly expressed and differentially expressed genes were analyzed for the presence of functional domains using InterPro (93) and PFAM (94). The presence of transmembrane helices and signal peptides was checked with the TMHMM server 2.0 (95) and SignalP 4.0 (96), respectively.

### Accession number(s).

*c*Eper1 sequencing data were deposited at the NCBI Sequence Read Archive under accession no. PRJEB13864.
