# Supplementary material for: Transcriptome Sequencing Reveals Novel Candidate Genes for Cardinium hertigii-Caused Cytoplasmic Incompatibility and Host-Cell Interaction
Source: mSystems. 2017 Nov 21;2(6):e00141-17. doi: 10.1128/mSystems.00141-17 (PMC5698495; doi:10.1128/mSystems.00141-17)
Supplement: TABLE S1 [file sys006172150st1.pdf]

Table S1. Potential novel *c*Eper1 transcripts and putative antisense RNAs, calculated with ReadXplorer. The parameter ‘number of read starts’ counts the number of reads and filters low coverage regions with a number of reads below a certain threshold. The threshold used to detect novel transcripts was set to 10 reads minimum. ’Coverage increase’ indicates the coverage increase in percent from one position to the neighboring one (for the detection of novel transcripts, the threshold was set to a minimum of 50% increase from the start of the transcript to the position before). ‘Transcript stop’ indicates the position at the chromosome where the putative transcript ends.

Position	Strand	Number of read starts	Coverage increase (%)	Transcript stop	Annotated *Cardinium* asRNA gene (locus_tag)	Sequence of the novel transcript
Chromosome						
24969	Fwd	17	114	25444	asRNA (CAHE_0024, putative UvrD-like DNA helicase)	ATAACAGCTTTACGCATGGTTGAAGATTCCACCAACTGCAAGACTTTTCCTACACCAAAATGAAGATGTTGGACAGTATGGCCAACCTGTATAGCACTAGGATCAGTAGGAATGCGTAGTCTAGCAGGTGCCACCATGGGGCCAATAGTGCGGATCGGTTTGTTGGACTGCTTTTGGGTGGCCATAGGCTTACCAGTATGGAGCGTATCCGTCGCTACCCTTTTAGCCATAGCTTTGGTTGCCGTATGGTCCATCCAACCCATCTCTTTTAAAAAACGACTTGGCTGAACATTGGCTAATTTGCCAAATCGGTATCTGCTTAATGCATAAGACAGCGTTAATTTTAGTTGGGCACGGGTAACAGCTACGTAAAGAAGACGCCTTTCTTCTTCTAAGTCTGCTTTGCTGCCCAACATCATAGCAGAAGGGAAGAGCTCTTCTTCCATGCCTACCAGGTATACGCATTTAAATTCTAG
149205	Fwd	28	216	149259		ATACTAGAGGTAATACTTACAACACCTCGCATGCTTACATTGGATTTAATCTTCC
149249	Rev	322	169	148780		GGGGATGGCCATGGGGCTCTATTTAAAGTTTAATAGCTAACGCAGCATAAAGGCGCTACCTGATTAAGTGGAGCTGGAGGGAATCGAACCCTCGTCCGAACAAGGAAACCACAACGCTTTCTACATGCTTAGCAACTGTTTATTGTCGGCGCAACCAAGGTCAGCCACTCACCTGATGTTACGCTTATGCCCGGTTATGATTTATGCGGCCATCACGCACCTGACCACACTAGTGCTATCCATCGATGCCCCTATGCTGATCCGATAACACAAAAACCAGCGGGACAAATTCCCCTTAATCTATCGATTAAGCAGCCATACGCAATTCATCGTCCTCTTCTTCTTCTTTTCGAAGCGCAACAAATTTGCAACGGCGGGAATTATTGATTACAGCTAAGTTGCCGTTTACGGATTGTGTAAGTTTTATACTAGAGGTAATACTTACAACACCTCGCATGCTTACATTGGAT
260920	Rev	26	325	260680		TTCCCTATTCCTGTATAGGCCTTTAAAAGGGTTCCTTAAGATAGGAAGCCTATCGTTAACGCCTTGGCGTGGCCTAAATAGATGGTTAGGTAGGGCTAGCTGCTACTCAATAATTTCATTATCTCAATGTTATAGGCAGCAGATGCATAAGAGAAAGCAGAATTTCCGGATCTATCCATTATTTCCTTCTTTGCCTTTTTAGCTATCAACTCCCTAGCAGACTCTATATTATTGTGTATTA
269073	Fwd	33	236	269139	asRNA (CAHE_rRNA3)	AACTGTCTCACGACGTTCTGAACCCAGCTCGCGTGCCACTTTAATGGGCGAACAGCCCAACCCTTGG
269670	Fwd	15	125	269753	asRNA (CAHE_rRNA3)	TCGTTACACCATTCGTGCAGGTCGGAACTTACCCGACAAGGAATTTCGCTACCTTAGGACCGTCAAGGTTACGGCCGCCGTTTA

312654	Fwd	31	135	312789		TTTGATGGATAAAATATACATCAAACCCTGTTAAAAAGTTTTGAAAGCACTGTTTATAGATGACGCACTAAGCCATTTAGCACGGAATCTAAGGGGTGGCTTTTGTTTTTCCAACTGCAAGGAGCGTCTTCAAAAC
409381	Rev	69	141	409065		TTATGCGTTTTATAAGTCAGAACAAAGTATGCATACATTTTACTTGCTAAAGTAAAAACATTTGTTTCGCTAGATGGTTGTGTTAGGCTTGTGTAATTCAAAAGGAACCATTACATTAATAGTGGACACTATTGACACATAAATAAGAAATAGCAAACCCACAGCCAGCTAAAAACCAAAATAAGCTAAGGTATGATAGTTGTATTATGGACAACTGGCATCGCTAATGTAGTTGGCTTATTTTTGATTTTTTAAGATGTAGGTGCCACTTAGAAAAGGGCTAAATGAGGTAAAAGCAATAAAAAAGATAACGTAAT
548516	Rev	899	110	548390		TACCCTAATAAGCTTTGGTAAAACTGGCGCTCGAGGATGTTGGAGCGGATGAAAAAAACAAGCCGAGTATCGCGCTGCTGCAACCACTTTACCCTTGTTCCTTTCCAGGCCTGGGGGATTTAGTAGG
630807	Rev	15	250	631033	asRNA (CAHE_0614, putative exported protein of unknown function)	TACCTCGAAGACCCAGCCGCTATCTCATCAAAAATTACTACAACCAGATAGTGCGTATAGTTCCATTATATCTAGCCCAGAGAAAAAACAAGCAACGATTAAGAAACTTGCTGCGTTAGAACATGCCTTTAAAAGGTTAGCAAGTAATTCTCAAGAAGTACGAAAGCTATTATCACGTGAGATGTCATCCATACCACAACCATCATTCGATCGAATCTATAACAATC
723650	Fwd	33	132	724049	asRNA (CAHE_0705, protein of unknown funtion)	TACGGCTCCATAGTAGCGCTAAGCGCATATAGCTGTTTTGACAAACCGACCCCTCCAAATAACACTAACCATTCAGGTAATAAAAAAATTGAAAGAAGGTGGATCGAAAAATCTAGATGCTTCAACAAAAAATTGGCACAGCAACAAATGGAGCAGCAGCAACAAACGGAACAGTAACAGCAACAGAAAACGGAACAGAAACAAGACGATCTAAATAAACAAACAAAGAAATCAGGAAAGGAGGCATCTTAAATGAGAAAAATGCATCAAAGATAGTATAGTAGTAGAACTGACAGATAAAACTAGAAAAGTAAACGAACTACTAGATAAAGCTTTAACAAATAACACTATATAAGCTTATTGTTTTTTCCATTTCTTACTCTTTGGTGATTATAGTAAAAA
728318	Fwd	68	262	728367		TAATTAGTTTGGTATAGTGTGTTGTATACAGCATATTTTTTTGTTACGAG
860835	Fwd	20	100	860940		TAAATATAGATCCAAATCTACTAAAAGGGGTCATTTGACTCAAAAGCCCGGTTTGTCTGATTTTAGTTATTAAACCCTTGTTACCCAATTGCTTCTGTATGATCCT
Plasmid						
47119	Fwd	463	265	47231	asRNA (CAHE_p0052, protein of unknown function)	CTTTTGTGATCATAAGATTATTTTTAATCCCTTGGAAGGCTAATTCTTACATCTAATCTGGTTCCCAACCAAAATAAAATAAGAACTAGCTTTCTTTGGGAGGCTAGTTTTCT
32541	Rev	40	134	32406	asRNA(CAHE_p0036, protein of unknown function)	GCTATTTCTTATTTGATTTTGGTTGCGAATCCAGGTTAAATGTGAGGAATAGCCTTCTAAGGGATTAAACAACAAAAGTATGTTGACAAAAGAAAAAATCCTTGAAAAGGCAGGAGGAGAGGGACACCTCATCCGC
