# Supplementary material for: Transcriptome Sequencing Reveals Novel Candidate Genes for Cardinium hertigii-Caused Cytoplasmic Incompatibility and Host-Cell Interaction
Source: mSystems. 2017 Nov 21;2(6):e00141-17. doi: 10.1128/mSystems.00141-17 (PMC5698495; doi:10.1128/mSystems.00141-17)
Supplement: TABLE S3 [file sys006172150st3.pdf]

Table S3. 30 *c*Eper1 genes that were <u>highest transcribed in male wasps</u> are listed and sorted by the fold-change compared to their expression in female wasps. Genes meeting the DE criteria ($p < 0.05$ and multiple testing correction of FDR < 10%) are highlighted in bold.

Locus tags from Penz et al., 2012[1]	Current GenBank locus tag[2]	Description	Mean *c*Eper1 expression in males	Mean *c*Eper1 expression in females	Fold change	*p*-value	FDR value
CAHE_p0035	AL022_RS04060	hypothetical protein	450.19	158.52	0.352	0.152	0.490
CAHE_0088	AL022_RS00410	hypothetical protein	1.58	0.57	0.363	0.707	0.912
CAHE_0416	AL022_RS01910	hypothetical protein	7.29	2.93	0.402	0.186	0.528
CAHE_p0059	AL022_RS04155	hypothetical protein	409.06	180.09	0.44	0.369	0.735
-	AL022_RS02750	hypothetical protein	17.85	7.96	0.446	0.059	0.341
-	AL022_RS01010	hypothetical protein	7.08	3.32	0.47	0.235	0.588
CAHE_p0063	AL022_RS04170	transposase	1142.75	551.01	0.482	0.242	0.601
**CAHE_p0027**	**AL022_RS04035**	**hypothetical protein**	**235.71**	**117.67**	**0.499**	**0.001**	**0.052**
CAHE_0539	AL022_RS02470	hypothetical protein	9.83	4.96	0.505	0.156	0.496
CAHE_p0049	AL022_RS04115	hypothetical protein	1191.93	602.62	0.506	0.314	0.699
CAHE_p0037	AL022_RS04070	hypothetical protein	329	170.11	0.517	0.515	0.819
CAHE_0552	AL022_RS02525	hypothetical protein	16.61	8.66	0.521	0.132	0.462
-	AL022_RS04150	transposase	1846.87	965.05	0.523	0.335	0.721
-	AL022_RS03860	hypothetical protein	15.29	8.23	0.538	0.115	0.452
**CAHE_p0026**	**AL022_RS04030**	**RING domain containing protein**	**1491.23**	**816.33**	**0.547**	**0.001**	**0.062**
CAHE_0385	AL022_RS01775	hypothetical protein	48.04	26.65	0.555	0.019	0.282

CAHE_0663	AL022_RS03030	hypothetical protein	16.98	9.55	0.562	0.128	0.458
**CAHE_0544**	**AL022_RS02490**	**hypothetical protein**	**459.68**	**259.51**	**0.565**	**0.002**	**0.095**
CAHE_0542	AL022_RS02485	hypothetical protein	465.55	264.74	0.569	0.043	0.312
CAHE_0605	AL022_RS02755	membrane protein	58.74	33.44	0.569	0.032	0.293
CAHE_0755	AL022_RS03470	thioredoxin	592.61	338.89	0.572	0.003	0.114
CAHE_0735	AL022_RS03370	hypothetical protein	172.35	100.46	0.583	0.003	0.114
CAHE_p0051	AL022_RS04125	hypothetical protein	550.86	321.77	0.584	0.255	0.610
CAHE_0319	AL022_RS01445	hypothetical protein	95.22	56.62	0.595	0.019	0.282
CAHE_0718	AL022_RS03290	iron-dependent repressor	229.37	138.59	0.604	0.249	0.607
CAHE_0732	AL022_RS03355	hypothetical protein	309.35	187.25	0.605	0.009	0.217
CAHE_p0022	AL022_RS04010	hypothetical protein	57.41	34.91	0.608	0.175	0.518
CAHE_0666	AL022_RS03045	hypothetical protein	164.99	101.21	0.613	0.019	0.282
CAHE_0787	AL022_RS03655	integrase	246.82	152.3	0.617	0.005	0.16
CAHE_p0040	AL022_RS04085	hypothetical protein	846.49	524.29	0.619	0.145	0.483

[1] Penz T, Schmitz-Esser S, Kelly SE, Cass BN, Muller A, Woyke T, Malfatti SA, Hunter MS, Horn M. 2012. Comparative genomics suggests an independent origin of cytoplasmic incompatibility in Cardinium hertigii. PLoS Genet 8(10): e1003012. Genbank accession numbers: HE983995 and HE983996

[2] Genbank accession numbers: NC_018605.1 and NC_018606.1
