# Supplementary material for: Transcriptome Sequencing Reveals Novel Candidate Genes for Cardinium hertigii-Caused Cytoplasmic Incompatibility and Host-Cell Interaction
Source: mSystems. 2017 Nov 21;2(6):e00141-17. doi: 10.1128/mSystems.00141-17 (PMC5698495; doi:10.1128/mSystems.00141-17)
Supplement: TABLE S4 [file sys006172150st4.pdf]

Table S4. 30 cEper1 genes that were highest transcribed in female wasps are listed and sorted by the fold-change compared to their expression in male wasps. Genes meeting the DE criteria ($p < 0.05$ and multiple testing correction of FDR < 10%) are highlighted in bold.

Locus tags from Penz et al., 2012[1]	Current GenBank locus tag[2]	Description	Mean *c*Eper1 expression in males	Mean *c*Eper1 expression in females	Fold change	*p*-value	FDR value
CAHE_0238	AL022_RS01105	DNA gyrase subunit A	587	1244.15	2.12	0.002	0.114
CAHE_0680	AL022_RS03115	Inversin protein alternative isoform, putative	817.92	1670.68	2.043	0.051	0.338
CAHE_0478	AL022_RS02230	tRNA 2-thiouridine(34) synthase MnmA	188.77	366.76	1.943	0.003	0.114
CAHE_0512	AL022_RS02375	hypothetical protein	13.15	25.52	1.94	0.044	0.312
CAHE_0241	AL022_RS01120	peptide ABC transporter ATP-binding protein	245.38	470.75	1.918	0.016	0.269
**CAHE_0131**	**AL022_RS00605**	**30S ribosomal protein S5**	**353.56**	**673.17**	**1.904**	**0.000**	**0.022**
CAHE_0133	AL022_RS00615	50S ribosomal protein L6	199.95	377.85	1.89	0.003	0.114
**CAHE_0339**	**AL022_RS01565**	**DNA-directed RNA polymerase subunit beta**	**1783.38**	**3335.95**	**1.871**	**0.000**	**0.009**
**CAHE_0565**	**AL022_RS02585**	**transcription elongation factor GreA**	**141.55**	**264.19**	**1.866**	**0.001**	**0.052**
CAHE_0706	AL022_RS03235	hypothetical protein	655.26	1217.74	1.858	0.341	0.721
**CAHE_0130**	**AL022_RS00600**	**50S ribosomal protein L30**	**147.84**	**274.74**	**1.858**	**0.000**	**0.052**
CAHE_0428	AL022_RS01970	3-oxoacyl-ACP synthase	581.62	1073.42	1.846	0.007	0.191
CAHE_0240	AL022_RS01115	peptide ABC transporter substrate-binding protein	207.59	378.84	1.825	0.010	0.224
**-**	**AL022_RS01110**	**hypothetical protein**	**214.87**	**390.65**	**1.818**	**0.000**	**0.04**
**CAHE_0242**	**AL022_RS01125**	**peptide ABC transporter permease**	**212.17**	**384.43**	**1.812**	**0.000**	**0.04**

**CAHE_0475**	**AL022_RS02215**	**30S ribosomal protein S9**	**213.37**	**385.64**	**1.807**	**0.001**	**0.052**
**CAHE_0338**	**AL022_RS01560**	**rpoB**	**2191.78**	**3822.27**	**1.744**	**0.000**	**0.035**
**CAHE_0132**	**AL022_RS00610**	**50S ribosomal protein L18**	**111.66**	**192.77**	**1.726**	**0.001**	**0.079**
CAHE_0411	AL022_RS01890	CMP deaminase	18.71	32.24	1.723	0.204	0.546
**CAHE_0678**	**AL022_RS03105**	**hypothetical protein**	**155.67**	**268.09**	**1.722**	**0.001**	**0.079**
CAHE_0794	AL022_RS03690	pseudouridine synthase	114.04	191.85	1.682	0.056	0.338
**CAHE_0102**	**AL022_RS00470**	**hypothetical protein**	**446.65**	**750.46**	**1.68**	**0.001**	**0.052**
**CAHE_0335**	**AL022_RS01545**	**50S ribosomal protein L1**	**701.12**	**1160.67**	**1.655**	**0.001**	**0.057**
CAHE_0278	AL022_RS01260	hypothetical protein	96.04	158.32	1.648	0.205	0.546
-	AL022_RS04090	transposase	1255.14	2053.5	1.636	0.438	0.764
CAHE_0376	AL022_RS01730	pyridoxal biosynthesis protein	350.33	567.97	1.621	0.021	0.282
CAHE_0430	AL022_RS01980	cysteine desulfurase activator complex subunit	216.22	350.1	1.619	0.027	0.282
CAHE_0753	AL022_RS03460	peptide chain release factor 1	482.62	780.34	1.617	0.011	0.226
CAHE_0765	AL022_RS03525	acetyl-CoA synthetase	292.87	473.06	1.615	0.209	0.549
CAHE_0129	AL022_RS00595	50S ribosomal protein L15	270.99	437.06	1.613	0.005	0.168

[1] Penz T, Schmitz-Esser S, Kelly SE, Cass BN, Muller A, Woyke T, Malfatti SA, Hunter MS, Horn M. 2012. Comparative genomics suggests an independent origin of cytoplasmic incompatibility in Cardinium hertigii. PLoS Genet 8(10): e1003012. Genbank accession numbers: HE983995 and HE983996

[2] Genbank accession numbers: NC_018605.1 and NC_018606.1
