# Supplementary material for: Transcriptome Sequencing Reveals Novel Candidate Genes for Cardinium hertigii-Caused Cytoplasmic Incompatibility and Host-Cell Interaction
Source: mSystems. 2017 Nov 21;2(6):e00141-17. doi: 10.1128/mSystems.00141-17 (PMC5698495; doi:10.1128/mSystems.00141-17)
Supplement: TABLE S5 [file sys006172150st5.pdf]

## Table S5. Gene expression of transposases in the *Cardinium hertigii c*Eper1 genome.

| Locus tags from Penz et al., 2012[1] | Current GenBank locus tag[2] | Best blast hit (GenBank accession no.) | Amino acid identities to best blast hit (%) | Mean normalized counts of *c*Eper1 expression | | | | | |
|---|---|---|---|---|---|---|---|---|---|
| | | | | 1st male replicate | 2nd male replicate | 3rd male replicate | 1st female replicate | 2nd female replicate | 3rd female replicate |
| CAHE_0001 | AL022_RS00010 | IS6 family transposase *[Cardinium* endosymbiont of *Bemisia tabaci*] (WP_034576929.1) | 89 | 162.07 | 136.56 | 180.53 | 133.62 | 123.89 | 76.25 |
| CAHE_0031 | AL022_RS00155 | IS6 family transposase [*Cardinium* endosymbiont cBtQ1 of *Bemisia tabaci*] (CDG50390.1) | 88 | 298.33 | 287.31 | 287.29 | 276.91 | 255.99 | 208.35 |
| CAHE_0041 | AL022_RS00195 | Transposase [*Cardinium* endosymbiont of *Ixodes scapularis*] (ACZ36949.1) | 87 | 590.93 | 564.86 | 582.35 | 503.48 | 507.49 | 428.51 |
| CAHE_0085 | AL022_RS00395 | Transposase [*Cardinium* endosymbiont of *Ixodes scapularis*] (ACZ36949.1) | 87 | 609.57 | 542.70 | 614.38 | 515.10 | 466.45 | 399.51 |
| CAHE_0086 | AL022_RS00400 | IS256 family transposase [*Cardinium* endosymbiont of *Bemisia tabaci*] (WP_051602130.1) | 92 | 134.82 | 106.41 | 99.00 | 99.73 | 77.62 | 82.69 |
| CAHE_0089 | AL022_RS00415 | IS3 family transposase, ssgr IS150 [*Cardinium* endosymbiont cBtQ1 of *Bemisia tabaci*] (CDG49338.1) | 89 | 681.29 | 575.51 | 644.47 | 505.42 | 556.00 | 209.42 |
| CAHE_0090 | AL022_RS00420 | IS6 family transposase [*Cardinium* endosymbiont cBtQ1 of *Bemisia tabaci*] (CDG50390.1) | 96 | 67.41 | 70.94 | 103.85 | 61.97 | 61.20 | 37.59 |
| CAHE_0179 | AL022_RS00820 | ISNCY family transposase [*Cardinium* endosymbiont cBtQ1 of *Bemisia tabaci*] ( CDG50399.1) | 81 | 269.65 | 354.70 | 395.03 | 303.06 | 372.41 | 331.85 |
| CAHE_0284 | AL022_RS01290 | IS6 family transposase [*Cardinium* endosymbiont cBtQ1 of *Bemisia tabaci*] (CDG50390.1) | 88 | 73.15 | 78.03 | 75.71 | 65.84 | 37.32 | 40.81 |
| CAHE_0285 | AL022_RS01295 | IS6 family transposase [*Cardinium* endosymbiont cBtQ1 of *Bemisia tabaci*] ( CDG50390.1) | 86 | 499.13 | 593.24 | 710.47 | 513.16 | 535.85 | 292.12 |
| CAHE_0370 | AL022_RS01695 | IS3 family transposase, ssgr IS150 [*Cardinium* endosymbiont cBtQ1 of *Bemisia tabaci*] (CDG49338.1) | 90 | 687.02 | 651.77 | 644.47 | 559.64 | 583.62 | 264.19 |
| CAHE_0447 | AL022_RS02080 | IS5 family transposase [*Cardinium* endosymbiont of *Bemisia tabaci*] (WP_034576548.1) | 90 | 71.71 | 63.85 | 79.59 | 60.03 | 60.45 | 45.11 |

| | | | | | | | | |
|---|---|---|---|---|---|---|---|---|
| CAHE_0471 | AL022_RS02195 | Hypothetical protein [*Cardinium* endosymbiont of *Bemisia tabaci*] (WP_034577391.1) | 88 | 281.12 | 283.76 | 311.56 | 224.63 | 221.66 | 121.36 |
| CAHE_0500 | AL022_RS02325 | Transposase [*Cardinium* endosymbiont of *Ixodes scapularis*] (ACZ36949.1) | 83 | 1041.29 | 924.89 | 920.12 | 759.09 | 753.78 | 651.89 |
| CAHE_0515 | AL022_RS02385 | Transposase [*Cardinium* endosymbiont of *Bemisia tabaci*] (WP_034577235.1) | 89 | 38.73 | 26.60 | 28.15 | 28.08 | 35.08 | 33.29 |
| CAHE_0516 | AL022_RS02390 | IS5 family transposase [*Cardinium* endosymbiont of *Bemisia tabaci*] (WP_051602192.1) | 88 | 43.03 | 54.09 | 71.82 | 56.16 | 48.51 | 59.07 |
| CAHE_0517 | AL022_RS02395 | IS5 family transposase [*Cardinium* endosymbiont of *Bemisia tabaci*] (WP_051602251.1) | 91 | 177.85 | 173.80 | 193.15 | 161.69 | 147.02 | 147.13 |
| CAHE_0539 | AL022_RS02470 | Hypothetical protein [*Cardinium* endosymbiont of *Bemisia tabaci*] (WP_051602225.1) | 89 | 4.30 | 10.64 | 14.56 | 3.87 | 6.72 | 4.30 |
| CAHE_0540 | AL022_RS02475 | Transposase [*Cardinium* endosymbiont of *Bemisia tabaci*] (WP_034576632.1) | 56 | 347.10 | 403.47 | 431.91 | 417.31 | 330.62 | 224.46 |
| CAHE_0541 | AL022_RS02480 | IS3 family transposase, ssgr IS150 [*Cardinium* endosymbiont cBtQ1 of *Bemisia tabaci*] (CDG49338.1) | 90 | 758.74 | 693.44 | 753.18 | 614.83 | 645.56 | 292.12 |
| CAHE_0542 | AL022_RS02485 | Hypothetical protein [*Candidatus* Amoebophilus asiaticus] (WP_012473024.1) | 42 | 456.10 | 451.36 | 489.18 | 323.39 | 380.62 | 90.21 |
| CAHE_0547 | AL022_RS02505 | IS3 family transposase, ssgr IS150 [*Cardinium* endosymbiont cBtQ1 of *Bemisia tabaci*] (CDG49338.1) | 90 | 777.38 | 707.63 | 689.12 | 597.40 | 553.02 | 254.53 |
| CAHE_0551 | AL022_RS02520 | Transposase [*Rickettsia parkeri* str. Portsmouth] (AFC74898.1) | 75 | 917.94 | 978.09 | 978.35 | 963.39 | 905.28 | 976.23 |
| CAHE_0553 | AL022_RS02530 | Hypothetical protein [*Cardinium* endosymbiont of *Bemisia tabaci*] (WP_034576733.1) | 83 | 484.79 | 521.41 | 552.26 | 420.21 | 404.50 | 205.13 |
| CAHE_0554 | AL022_RS02535 | Tyrosine recombinase XerD [*Legionella nautarum*] (KTD32257.1) | 87 | 67.41 | 59.41 | 59.21 | 66.81 | 63.44 | 53.70 |
| CAHE_0594 | AL022_RS02710 | Hypothetical protein [*Cardinium* endosymbiont of *Bemisia tabaci*] (WP_034576733.1) | 81 | 1580.59 | 1566.01 | 1713.09 | 1463.00 | 1215.00 | 750.70 |
| CAHE_0601 | AL022_RS02735 | IS5 family transposase [*Cardinium* endosymbiont of *Bemisia tabaci*] (WP_051602192.1) | 95 | 48.77 | 76.26 | 66.97 | 70.68 | 67.17 | 60.14 |
| CAHE_0602 | AL022_RS02740 | IS5 family transposase [*Cardinium* endosymbiont of *Bemisia tabaci*] (WP_034576548.1) | 90 | 73.15 | 45.22 | 55.32 | 69.71 | 53.73 | 61.22 |
| CAHE_0609 | AL022_RS02775 | ISNCY family transposase [*Cardinium* endosymbiont cBtQ1 of *Bemisia tabaci*] (CDG50399.1) | 87 | 34.42 | 39.90 | 56.29 | 31.95 | 37.32 | 17.18 |
| CAHE_0610 | AL022_RS02780 | IS5 family transposase [*Cardinium* endosymbiont of *Bemisia tabaci*] (WP_034576548.1) | 90 | 84.62 | 66.51 | 91.24 | 67.78 | 58.21 | 60.14 |

| | | | | | | | | | |
|---|---|---|---|---|---|---|---|---|---|
| CAHE_0611 | AL022_RS02785 | Hypothetical protein [*Cardinium* endosymbiont of *Bemisia tabaci*] (WP_034576570.1) | 90 | 60.24 | 72.71 | 65.03 | 78.43 | 67.91 | 75.18 |
| CAHE_0627 | AL022_RS02865 | IS6 family transposase [*Cardinium* endosymbiont of *Bemisia tabaci*] (WP_034576929.1) | 89 | 187.89 | 190.65 | 255.26 | 185.90 | 194.04 | 104.17 |
| CAHE_0631 | AL022_RS02885 | Transposase [*Cardinium* endosymbiont of *Ixodes scapularis*] (ACZ36949.1) | 83 | 1061.37 | 992.28 | 858.97 | 766.84 | 731.39 | 623.97 |
| CAHE_0682 | AL022_RS03130 | Transposase [*Candidatus* Amoebophilus asiaticus] (ADL32106.1) | 78 | 68.85 | 58.53 | 82.50 | 49.38 | 46.27 | 39.74 |
| CAHE_0683 | AL022_RS03135 | Hypothetical protein [*Acinetobacter* sp. CIP 102529] (WP_004763135.1) | 65 | 51.63 | 33.70 | 32.03 | 28.08 | 34.33 | 42.96 |
| CAHE_0686 | AL022_RS03140 | IS3 family transposase, ssgr IS150 [*Cardinium* endosymbiont cBtQ1 of *Bemisia tabaci*] (CDG49338.1) | 90 | 695.63 | 612.75 | 658.06 | 543.18 | 515.70 | 220.16 |
| CAHE_0688 | AL022_RS03145 | ISNCY family transposase [*Cardinium* endosymbiont cBtQ1 of *Bemisia tabaci*] (CDG50399.1) | 86 | 787.42 | 813.16 | 801.71 | 1309.05 | 540.33 | 652.97 |
| CAHE_0689 | AL022_RS03150 | Transposase [*Cardinium* endosymbiont of *Bemisia tabaci*] (WP_034577235.1) | 88 | 30.12 | 31.04 | 33.97 | 55.19 | 26.87 | 19.33 |
| CAHE_0708 | AL022_RS03240 | Transposase [*Rickettsia felis* URRWXCal2] (AAY60952.1) | 75 | 735.79 | 669.50 | 715.32 | 583.84 | 617.20 | 640.08 |
| CAHE_0738 | AL022_RS03385 | IS110 family transposase [*Ornithobacterium rhinotracheale*] (WP_014790141.1) | 51 | 1662.34 | 1499.51 | 1551.97 | 1185.11 | 1235.15 | 1229.68 |
| CAHE_0739 | AL022_RS03390 | IS256 family transposase [*Cardinium* endosymbiont of *Bemisia tabaci*] (WP_051602183.1) | 88 | 309.81 | 253.61 | 264.00 | 314.67 | 235.84 | 241.64 |
| CAHE_0787 | AL022_RS03655 | IS6 family transposase [*Cardinium* endosymbiont cBtQ1 of *Bemisia tabaci*] (CDG50390.1) | 88 | 246.70 | 228.78 | 264.97 | 201.39 | 147.02 | 108.47 |
| CAHE_0809 | AL022_RS03760 | Hypothetical protein [*Cardinium* endosymbiont of *Bemisia tabaci*] (WP_051602146.1) | 96 | 259.61 | 215.48 | 219.35 | 243.99 | 217.18 | 123.51 |
| CAHE_0810 | AL022_RS03765 | IS3 family transposase, ssgr IS150 [*Cardinium* endosymbiont cBtQ1 of *Bemisia tabaci*] (CDG49338.1) | 90 | 742.96 | 712.95 | 699.79 | 602.24 | 598.54 | 279.23 |
| CAHE_0811 | AL022_RS03770 | IS3 family transposase, ssgr IS150 [*Cardinium* endosymbiont cBtQ1 of *Bemisia tabaci*] (CDG50385.1) | 88 | 489.09 | 462.89 | 492.09 | 383.42 | 387.34 | 143.91 |
| CAHE_0816 | AL022_RS03795 | Transposase [*Rickettsia parkeri* str. Portsmouth] (AFC74898.1) | 74 | 598.10 | 578.17 | 608.56 | 529.62 | 660.49 | 561.68 |
| CAHE_0818 | AL022_RS03805 | Resolvase [*Cardinium* endosymbiont cBtQ1 of *Bemisia tabaci*] (CDG50400.1) | 85 | 500.57 | 485.94 | 499.85 | 397.94 | 323.15 | 253.45 |
| CAHE_0823 | AL022_RS03825 | IS110 family transposase [*Ornithobacterium rhinotracheale*] (WP_014790141.1) | 51 | 1626.48 | 1570.45 | 1526.73 | 1230.62 | 1263.51 | 1101.88 |
| CAHE_0824 | AL022_RS03830 | Resolvase [*Enterobacter cloacae* complex sp. GN02141] (WP_047721425.1) | 77 | 238.09 | 231.44 | 231.97 | 174.28 | 165.68 | 138.54 |

| | | | | | | | | | |
|---|---|---|---|---|---|---|---|---|---|
| CAHE_0826 | AL022_RS03835 | DDE transposase [*Salmonella enterica*] (WP_001525361.1) | 77 | 76.02 | 46.11 | 54.35 | 45.51 | 28.36 | 50.48 |
| CAHE_0827 | AL022_RS03840 | IS110 family transposase [*Ornithobacterium rhinotracheale*] (WP_014790141.1) | 51 | 1659.47 | 1536.75 | 1551.97 | 1204.48 | 1289.63 | 1169.54 |
| CAHE_0828 | AL022_RS03845 | Integrase [*Legionella pneumophila*] (WP_061504282.1) | 78 | 45.90 | 24.83 | 42.71 | 18.40 | 20.90 | 34.37 |
| CAHE_0830 | AL022_RS03850 | Hypothetical protein [*Cardinium* endosymbiont of *Bemisia tabaci*] (WP_034576733.1) | 81 | 1207.67 | 1239.69 | 1327.76 | 1058.28 | 1024.69 | 592.82 |
| CAHE_0831 | AL022_RS03855 | IS6 family transposase [*Cardinium* endosymbiont of *Bemisia tabaci*] (WP_034576929.1) | 89 | 504.87 | 555.11 | 560.03 | 425.05 | 388.83 | 262.05 |
| CAHE_0833 | AL022_RS03865 | IS3 family transposase, ssgr IS150 [*Cardinium* endosymbiont cBtQ1 of *Bemisia tabaci*] (CDG50385.1) | 88 | 691.33 | 670.39 | 728.91 | 570.29 | 568.69 | 266.34 |
| CAHE_p0001 | AL022_RS03915 | IS110 family transposase [*Ornithobacterium rhinotracheale*] (WP_014790141.1) | 51 | 1917.64 | 1740.71 | 1721.82 | 1337.13 | 1385.91 | 1341.37 |
| CAHE_p0002 | AL022_RS03920 | ISNCY family transposase [*Cardinium* endosymbiont cBtQ1 of *Bemisia tabaci*] (CDG50399.1) | 76 | 463.28 | 504.57 | 549.35 | 502.51 | 305.24 | 329.71 |
| CAHE_p0003 | AL022_RS03925 | Transposase [*Cardinium* endosymbiont of *Ixodes scapularis*] (ACZ36949.1) | 78 | 1904.74 | 1672.43 | 1577.21 | 1435.89 | 1332.92 | 1336.00 |
| CAHE_p0004 | AL022_RS03930 | Integrase [*Legionella pneumophila*] (WP_042238319.1) | 79 | 193.63 | 174.69 | 192.18 | 167.50 | 151.50 | 165.39 |
| CAHE_p0005 | AL022_RS03935 | Hypothetical protein [*Cardinium* endosymbiont of *Bemisia tabaci*] (WP_034577172.1) | 79 | 1150.30 | 1226.39 | 1406.38 | 1240.30 | 915.73 | 719.55 |
| CAHE_p0006 | AL022_RS03940 | IS6 family transposase [*Cardinium* endosymbiont of *Bemisia tabaci*] (WP_014934436.1) | 90 | 1364.01 | 1374.48 | 1573.32 | 1213.19 | 1155.30 | 768.95 |
| CAHE_p0012 | AL022_RS03960 | Resolvase [*Enterobacter cloacae* complex sp. GN02141] (WP_047721425.1) | 77 | 238.09 | 231.44 | 231.97 | 174.28 | 165.68 | 138.54 |
| CAHE_p0013 | AL022_RS03965 | Hypothetical protein [*Photorhabdus temperata*] (WP_021325327.1) | 36 | 1120.18 | 1150.13 | 1324.85 | 1664.39 | 825.42 | 712.03 |
| CAHE_p0032 | AL022_RS04050 | Hypothetical protein CHV_p007 [*Cardinium* endosymbiont cBtQ1 of *Bemisia tabaci*] (CDG50372.1) | 77 | 78.89 | 2.66 | 1.94 | 67.78 | 2.24 | 9.67 |
| CAHE_p0039 | AL022_RS04080 | Hypothetical protein [*Cardinium* endosymbiont of *Bemisia tabaci*] (WP_034577391.1) | 80 | 958.11 | 201.29 | 237.79 | 1058.28 | 165.68 | 563.83 |
| CAHE_p0040 | AL022_RS04085 | IS256 family transposase [*Cardinium* endosymbiont of *Bemisia tabaci*] (WP_051602183.1) | 88 | 476.18 | 1028.64 | 1034.65 | 405.69 | 778.41 | 388.77 |
| CAHE_p0046 | AL022_RS04105 | IS6 family transposase [*Cardinium* endosymbiont of *Bemisia tabaci*] (WP_034576929.1) | 87 | 1287.99 | 524.07 | 720.18 | 1195.76 | 641.83 | 745.33 |
| CAHE_p0047 | AL022_RS04110 | IS110 family transposase [*Ornithobacterium rhinotracheale*] (WP_014790141.1) | 51 | 1682.42 | 1324.82 | 1576.23 | 1255.79 | 1029.17 | 1188.87 |

| | | | | | | | | | |
|---|---|---|---|---|---|---|---|---|---|
| CAHE_p0062 | AL022_RS04165 | Hypothetical protein [*Cardinium* endosymbiont of *Bemisia tabaci*] (WP_034576556.1) | 84 | 1501.70 | 15.96 | 14.56 | 1344.87 | 8.96 | 581.01 |
| CAHE_p0063 | AL022_RS04170 | IS6 family transposase [*Cardinium* endosymbiont cBtQ1 of *Bemisia tabaci*] (CDG50390.1) | 88 | 384.39 | 1456.94 | 1586.91 | 342.75 | 1154.55 | 155.72 |
| CAHE_p0064 | AL022_RS04175 | IS3 family transposase, ssgr IS150 [*Cardinium* endosymbiont cBtQ1 of *Bemisia tabaci*] (CDG50385.1) | 88 | 3336.16 | 342.29 | 386.29 | 2652.95 | 303.00 | 1115.84 |
| CAHE_p0065 | AL022_RS04180 | Transposase [*Rickettsia parkeri* str. Portsmouth] (AFC74898.1) | 80 | 1141.69 | 3035.37 | 3137.91 | 1117.34 | 2709.12 | 1007.37 |

[1] Penz T, Schmitz-Esser S, Kelly SE, Cass BN, Muller A, Woyke T, Malfatti SA, Hunter MS, Horn M. 2012. Comparative genomics suggests an independent origin of cytoplasmic incompatibility in Cardinium hertigii. PLoS Genet 8(10): e1003012. Genbank accession numbers: HE983995 and HE983996

[2] Genbank accession numbers: NC_018605.1 and NC_018606.1
