# Supplementary material for: Transcriptome Sequencing Reveals Novel Candidate Genes for Cardinium hertigii-Caused Cytoplasmic Incompatibility and Host-Cell Interaction
Source: mSystems. 2017 Nov 21;2(6):e00141-17. doi: 10.1128/mSystems.00141-17 (PMC5698495; doi:10.1128/mSystems.00141-17)
Supplement: TABLE S6 [file sys006172150st6.pdf]

Table S6. Transcription of *Cardinium hertigii c*Eper1 genes encoding proteins possibly involved in host cell interactions.

				Mean normalized counts of *c*Eper1 expression					
**Locus tags from Penz et al., 2012[1]**	**Current GenBank locus tag[2]**	**Best blast hit (GenBank accession no.)**	**Amino acid identities to best blast hit in %**	**1st male replicate**	**2nd male replicate**	**3rd male replicate**	**1st female replicate**	**2nd female replicate**	**3rd female replicate**
**Ankyrin repeat proteins**									
CAHE_0040	AL022_RS00190	Hypothetical protein [*Cardinium* endosymbiont of *Bemisia tabaci*] (WP_034576299.1)	30	229.49	164.94	175.68	236.25	201.51	123.51
CAHE_0396	AL022_RS01830	Hypothetical protein *[Cardinium* endosymbiont of *Bemisia tabaci*] (WP_051602196.1)	41	301.20	284.65	293.12	229.47	193.30	221.24
CAHE_0435	AL022_RS02005	Hypothetical protein *[Cardinium* endosymbiont of *Bemisia tabaci*] (WP_034576647.1)	66	2071.11	1808.10	1813.06	1550.14	1569.50	1053.55
CAHE_0397	AL022_RS01835	Hypothetical protein [*Oidiodendron maius* Zn] (KIM99444.1)	58	163.51	167.60	187.32	143.30	110.45	115.99
CAHE_0491	AL022_RS02295	Ankyrin repeats-containing protein *[Cardinium* endosymbiont cBtQ1 of *Bemisia tabaci]* (CDG49464.1)	73	361.44	393.72	394.06	303.06	261.96	370.52
CAHE_0588	AL022_RS02685	Hypothetical protein *[Cardinium* endosymbiont of *Bemisia tabaci]* (WP_034576449.1)	86	153.47	172.03	158.21	169.44	234.34	219.09
CAHE_0670	AL022_RS03065	Hypothetical protein *[Cardinium* endosymbiont of *Bemisia tabaci*] (WP_034576875.1)	63	167.81	184.45	167.91	213.01	238.07	328.63
CAHE_0834	AL022_RS03870	Hypothetical protein *[Cardinium* endosymbiont of *Bemisia tabaci]* (WP_034576747.1)	80	1055.64	1011.79	992.91	823.96	778.41	962.27
CAHE_p0007	AL022_RS03945	Hypothetical protein [*Rickettsia bellii*] (WP_011477011.1)	46	1808.64	1882.59	2135.29	1338.09	1359.04	1222.16
CAHE_p0014	AL022_RS03970	Ankyrin repeat protein [*Trichomonas vaginalis* G3] (XP_001316939.1)	41	1691.03	1806.33	1835.38	1116.37	1305.31	982.67
CAHE_p0019	AL022_RS03995	Hypothetical protein [*Cardinium* endosymbiont of *Bemisia tabaci*] WP_051602196.1	43	130.52	161.39	163.06	112.31	141.80	75.18
CAHE_0680	AL022_RS03115	Hypothetical protein *[Cardinium* endosymbiont of *Bemisia tabaci]* (WP_034576855.1)	88	948.07	828.23	677.47	1098.94	1407.55	2505.54
CAHE_p0026	AL022_RS04030	Hypothetical protein [*Cardinium* endosymbiont of *Bemisia tabaci*] (WP_034577639.1)	48	1257.87	1475.57	1740.26	1005.02	920.95	523.02
CAHE_0095	AL022_RS00440	Ankyrin repeats-containing protein *[Cardinium* endosymbiont cBtQ1 of *Bemisia tabaci*] (CDG49910.1)	93	1041.29	819.36	969.62	767.81	714.22	855.94

**TPR repeat proteins**									
CAHE_0312	AL022_RS01410	Hypothetical protein *[Cardinium* endosymbiont of *Bemisia tabaci*] (WP_034577424.1)	88	566.54	462.00	515.38	454.10	421.67	465.02
CAHE_0450	AL022_RS02095	Hypothetical protein [*Cardinium* endosymbiont of *Bemisia tabaci*] (WP_034577415.1)	80	302.63	298.84	351.35	285.63	238.07	224.46
CAHE_0452	AL022_RS02105	Hypothetical protein *[Cardinium* endosymbiont of *Bemisia tabaci*] (WP_051602092.1)	83	226.62	219.92	201.88	188.80	231.36	158.95
**Ubiquitin protease**									
CAHE_0028	AL022_RS00135	Ubiquitin specific protease [*Cardinium* endosymbiont cBtQ1 of *Bemisia tabaci*] (CDG50083.1)	46	621.05	654.43	636.71	756.19	792.59	895.68
**General host cell interaction genes**									
CAHE_0010	AL022_RS00050	Hypothetical protein [*Cardinium* endosymbiont of *Bemisia tabaci*] (WP_034576461.1)	46	275.38	219.03	231.00	258.52	303.75	287.82
CAHE_0286	AL022_RS01300	Patatin [*Wolbachia* endosymbiont of *Culex quinquefasciatus*] (WP_007302998.1)	64	460.41	498.36	508.59	481.21	535.11	234.12
CAHE_0706	AL022_RS03235	Hypothetical protein [*Bacillus cereus* VD136] (EOP51398.1)	87	711.41	714.73	539.65	2247.26	435.10	970.86
CAHE_0677	AL022_RS03100	DEAD/DEAH box helicase [*Cardinium* endosymbiont of *Bemisia tabaci*] (WP_034576864.1)	97	3985.89	4329.15	3868.76	4571.98	5379.44	7352.31
CAHE_0017	AL022_RS00085	Hypothetical protein [*Cardinium* endosymbiont of *Bemisia tabaci*] (WP_034576476.1)	88	760.17	790.99	719.21	872.38	971.70	1141.62
CAHE_0267	AL022_RS01210	DNA mismatch repair protein mutS [*Cardinium* endosymbiont cBtQ1 of *Bemisia tabaci*] (CDG49367.1)	92	1147.43	1004.70	1088.03	963.39	1079.17	961.19
CAHE_0050	AL022_RS00235	Hypothetical protein [*Cardinium* endosymbiont of *Bemisia tabaci*] (WP_034577189.1)	68	5738.59	6495.50	6406.85	6171.50	7742.27	6147.33

[1] Penz T, Schmitz-Esser S, Kelly SE, Cass BN, Muller A, Woyke T, Malfatti SA, Hunter MS, Horn M. 2012. Comparative genomics suggests an independent origin of cytoplasmic incompatibility in Cardinium hertigii. PLoS Genet 8(10): e1003012. Genbank accession numbers: HE983995 and HE983996

[2] Genbank accession numbers: NC_018605.1 and NC_018606.1
