# Supplementary figures and images for: Transcriptome Sequencing Reveals Novel Candidate Genes for Cardinium hertigii-Caused Cytoplasmic Incompatibility and Host-Cell Interaction
Source: mSystems. 2017 Nov 21;2(6):e00141-17. doi: 10.1128/mSystems.00141-17 (PMC5698495; doi:10.1128/mSystems.00141-17)

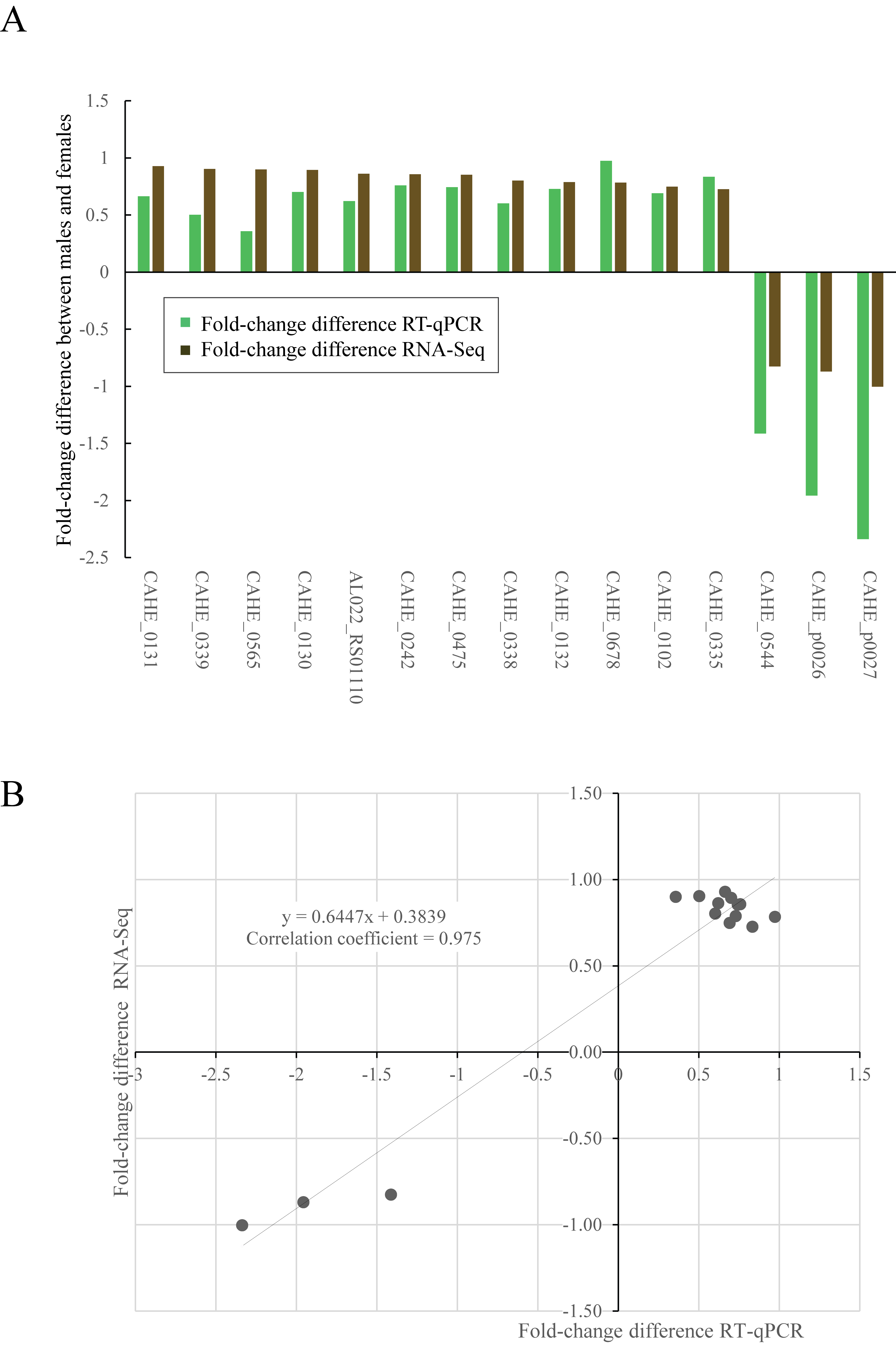

Supplement: FIG S1 [file sys006172150sf10.tif]
