# Supplementary material for: Transcriptome Sequencing Reveals Novel Candidate Genes for Cardinium hertigii-Caused Cytoplasmic Incompatibility and Host-Cell Interaction
Source: mSystems. 2017 Nov 21;2(6):e00141-17. doi: 10.1128/mSystems.00141-17 (PMC5698495; doi:10.1128/mSystems.00141-17)
Supplement: TABLE S7 [file sys006172150st7.pdf]

Table S7. Transcription of *Cardinium hertigii c*Eper1 genes encoding proteins of the putative antifeeding prophage-like secretion system.

				Mean normalized counts of *c*Eper1 expression					
**Locus tags from Penz et al., 2012[1]**	**Current GenBank locus tag[2]**	**Best blast hit (GenBank accession no.)**	**Amino acid identities to best blast hit in %**	**1st male replicate**	**2nd male replicate**	**3rd male replicate**	**1st female replicate**	**2nd female replicate**	**3rd female replicate**
CAHE_0036	AL022_RS00170	Adhesin *Cardinium* endosymbiont of *Bemisia tabaci* (WP_034576687.1)	97	684.16	574.62	589.15	624.51	643.32	545.57
CAHE_0037	AL022_RS00175	Hypothetical protein *Cardinium* endosymbiont of *Bemisia tabaci* (WP_034576687.1)	94	1580.59	1419.7	1305.44	1281.94	1357.55	1125.51
CAHE_0118	AL022_RS00550	Hypothetical protein *Cardinium* endosymbiont of *Bemisia tabaci* (WP_034576687.1)	83	625.35	554.22	529.94	455.07	533.62	639
CAHE_0409	AL022_RS01880	Peptidase M41 *Cardinium* endosymbiont of *Bemisia tabaci* (WP_034576687.1)	94	560.81	486.83	433.85	634.19	710.49	898.9
CAHE_0456	AL022_RS02120	Hypothetical protein *Cardinium* endosymbiont of *Bemisia tabaci* (WP_034576687.1)	96	309.81	245.63	242.65	237.22	257.48	225.53
CAHE_0457	AL022_RS02125	Hypothetical protein *Cardinium* endosymbiont of *Bemisia tabaci* (WP_034576687.1)	87	448.93	392.83	357.18	405.69	400.77	424.21
CAHE_0458	AL022_RS02130	Phage tail protein *Cardinium* endosymbiont of *Bemisia tabaci* (WP_034576687.1)	98	3402.13	2422.62	2556.53	3907.78	3536.04	2710.67
CAHE_0459	AL022_RS02135	Hypothetical protein *Cardinium* endosymbiont of *Bemisia tabaci* (WP_034576687.1)	95	648.3	381.31	450.35	660.33	656.01	326.48
CAHE_0460	AL022_RS02140	Hypothetical protein *Cardinium* endosymbiont of *Bemisia tabaci* (WP_034576687.1)	93	570.85	386.63	391.15	583.84	582.87	279.23

CAHE_0461	AL022_RS02145	Hypothetical protein *Cardinium* endosymbiont of *Bemisia tabaci* (WP_034576687.1)	93	502	312.14	313.5	516.07	479.88	265.27
CAHE_0463	AL022_RS02150	Hypothetical protein *Cardinium* endosymbiont of *Bemisia tabaci* (WP_034576687.1)	84	1125.92	799.86	644.47	1209.32	1176.94	1008.45
CAHE_0760	AL022_RS03495	Hypothetical protein *Cardinium* endosymbiont of *Bemisia tabaci* (WP_034576687.1)	91	787.42	582.6	628.94	679.7	699.3	591.75
CAHE_0761	AL022_RS03500	Hypothetical protein *Cardinium* endosymbiont of *Bemisia tabaci* (WP_034576687.1)	93	137.69	149.86	162.09	165.57	138.81	149.28
CAHE_0762	AL022_RS03505	Type VI secretion protein *Cardinium* endosymbiont of *Bemisia tabaci* (WP_034576687.1)	95	192.19	203.95	193.15	204.3	147.02	313.6
CAHE_0763	AL022_RS03510	Hypothetical protein *Cardinium* endosymbiont of *Bemisia tabaci* (WP_034576687.1)	96	530.69	497.47	438.71	415.37	409.73	567.05

[1] Penz T, Schmitz-Esser S, Kelly SE, Cass BN, Muller A, Woyke T, Malfatti SA, Hunter MS, Horn M. 2012. Comparative genomics suggests an independent origin of cytoplasmic incompatibility in Cardinium hertigii. PLoS Genet 8(10): e1003012. Genbank accession numbers: HE983995 and HE983996

[2] Genbank accession numbers: NC_018605.1 and NC_018606.1
