# Supplementary material for: Transcriptome Sequencing Reveals Novel Candidate Genes for Cardinium hertigii-Caused Cytoplasmic Incompatibility and Host-Cell Interaction
Source: mSystems. 2017 Nov 21;2(6):e00141-17. doi: 10.1128/mSystems.00141-17 (PMC5698495; doi:10.1128/mSystems.00141-17)
Supplement: TABLE S8 [file sys006172150st8.pdf]

Table S8. Primer sequences, amplicon length and annealing temperature of primers targeting *c*Eper1 DE genes and two housekeeping genes (*gyrB* and *groEL*). Annealing temperatures were optimized with genomic DNA isolated from a culture of *E. suzannae* infected with *Cardinium hertigii c*Eper1.

***Cardinium* locus tag[1]**	**Forward primer sequence (5' - 3')**	**Reverse primer sequence (5' - 3')**	**Expected length of amplificate [bp]**	**Annealing temperature [°C]**
AL022_RS02415- *GyrB*	ACGAGACCCTACGCTTTGTG	GCTACACCCAGTGCAGTGAT	195	57
AL022_RS01165- *GroEL*	TTTGAGGCATAGGAGCAGGC	CCATTGTAGCCAATGCAGGC	249	57
CAHE_0339	CATAGAGCGTGGGGTAGCAG	AACGGCCATTTGATCCCCAT	250	57
CAHE_0131	TGCGGATTAGAAGAACCCTGG	GTAGAGGCAGCTAAGCGCAA	191	58
CAHE_0338	GCTAAGGTAGTGCCTGCCAA	TCCAATCCTGGACCACTTGC	197	57
AL022_RS01110	TCCGTTCTAGCCCCATACCA	TTGACCCAAATGATGGCCGA	213	57
CAHE_0242	GGCTATGTGCTCGTAGCAGT	AACCGTCATGGCTACTTCGG	174	57
CAHE_0130	TGGAACAAATTAGAATTACACAGGT	TGACTTTACGTACCATACCCCA	150	55
CAHE_0475	CCGCCGTAGCTAGGGTGTAT	ATACCCAAGCGAATGGCTTCA	188	58
CAHE_0565	GCCAATGACTTGGCAGAAGC	CAGCTTCACCCTGAGAGACA	250	57
CAHE_0102	TCTCATCTGCCATTGTGGCT	CAATAGCCCACCCCAAAGGT	225	57
CAHE_p0027	GGAAACTCGTTTACATCTCACACA	CACGCCCTGCTCCATTATCT	182	57
CAHE_0335	CGGTTCAGCTCGTGCAAAAA	CAGCTTGCCTAGCCTCTTGT	195	57
CAHE_p0026	GGGCTGCTAGAGGTGGTTTT	TGAGCATCATCTGGGGAAAGG	157	59
CAHE_0132	CCGCGTCTTTCGCTTTTCAA	TTTAGCCAACGCTTGTTGCG	171	57
CAHE_0678	GCACAGCTATGCAGGAAGGA	CCCAATACCGAATACGCCCA	151	57
CAHE_0544	GCTGCATAACTGATCCAAAGGG	AGCATCTTCTAAGTCCCAATCAGA	233	57

[1] Locus tags from Penz T, Schmitz-Esser S, Kelly SE, Cass BN, Muller A, Woyke T, Malfatti SA, Hunter MS, Horn M. 2012. Comparative genomics suggests an independent origin of cytoplasmic incompatibility in Cardinium hertigii. PLoS Genet 8(10): e1003012. (Genbank accession numbers: HE983995 and HE983996) and current GenBank locus tags (NC_018605.1 and NC_018606.1).
