# Supplementary material for: Transcriptome Sequencing Reveals Novel Candidate Genes for Cardinium hertigii-Caused Cytoplasmic Incompatibility and Host-Cell Interaction
Source: mSystems. 2017 Nov 21;2(6):e00141-17. doi: 10.1128/mSystems.00141-17 (PMC5698495; doi:10.1128/mSystems.00141-17)
Supplement: TABLE S9 [file sys006172150st9.pdf]

Table S9. MIQE guidelines for RT q-PCR assays

Item	Importance	Status	Remarks
**Experimental design**			
definition of experimental and control groups	E	OK	Male and female replicates
number within each group	E	OK	three biological replicates per sample
Assay carried out by core lab or investigator's lab?	D	OK	Investigator's lab
acknowledgement of authors' contributions	D	OK	done
**Sample**			
description	E	OK	RNA isolated from wasp pools
volume/mass of sample processed	D	OK	Between 350 and 500 wasps which were one to three days old were collected from each cage
microdissection or macrodissection	E	OK	macrodissection
processing procedure	E	OK	Trizol protocol
If frozen - how and how quickly?	E	OK	shock frozen at -80°C for two minutes
If fixed - with what, how quickly?	E	OK	samples were not fixed, TRIzol reagent added immediately after harvesting
sample storage conditions and duration	E	OK	from harvest until addition of TRIzol approximately 5min
**Nucleic acid extraction**			
procedure and/or instrumentation	E	OK	TRIzol-based total RNA extraction, mechanical lysis
name of kit and details of any modifications	E	OK	TRIzol reagent (Life Technologies)
source of additional reagents used	D	OK	-
details of DNase or RNase treatment	E	OK	TURBO DNA-free™ kit (Life Technologies)
contamination assessment (DNA or RNA)	E	OK	PCR and agarose gelelectrophoresis; –RT controls (reverse transcription reaction without addition of enzyme) analysed with qPCR
nucleic acid quantification	E	OK	done
instrument and method	E	OK	Agilent 2100 bioanalyzer (Agilent Technologies, Palo Alto, CA)
RNA integrity method/instrument	E		Agilent 2100 bioanalyzer (Agilent Technologies, Palo Alto, CA)
RIN/RQI or Cq of 3' and 5' transcripts	E		>4
electrophoresis traces	D		
inhibition testing (Cq dilutions, spike or other)	E		no inhibition
**Reverse transcription**			
Reaction conditions	E	OK	
Amount of RNA and reaction volume	E	OK	15µl
Priming oligonucleotide and concentration	E	OK	random hexamer primers (final concentration 8 ng μl^{-1})
Reverse transcriptase and concentration	E	OK	RevertAid H Minus First Strand cDNA Synthesis Kit, Thermo Scientific, Vienna, Austria
Temperature and time	E	OK	RNA secondary structures were broken up at 65°C for 5 min, cDNA synthesis was done at 45°C for 60

			min after a pre-incubation at 25°C for 5 min. The reaction was terminated by heating at 70°C for 5 min and cDNA was stored at -20°C.
Manufacturer of reagens and catalogue numbers	D	OK	Invitrogen
Cqs with and without RT	D	OK	below limit of detection
Storage conditions of cDNA	D	OK	-20°C for 2 weeks
**qPCR target information**		OK	
if multiplex, efficiency and LOD of each assay.	E	OK	no multiplexing
sequence accession number	E		CAHE_0131 Gene ID: 13654403<br>CAHE_0339 Gene ID: 13654411<br>CAHE_0565 Gene ID: 13654933<br>CAHE_0130 Gene ID: 13654389<br>AL022_RS01110 -<br>CAHE_0242 Gene ID: 13654084<br>CAHE_0475 Gene ID: 13654387<br>CAHE_0338 Gene ID: 13654386<br>CAHE_0132 Gene ID: 13654418<br>CAHE_0678 Gene ID: 13654064<br>CAHE_0102 Gene ID: 13654522<br>CAHE_0335 Gene ID: 13654385<br>CAHE_0544 Gene ID: 13654662<br>CAHE_p0026 Gene ID: 13654955<br>CAHE_p0027 Gene ID: 13654956
location of amplicon	D	OK	
amplicon length	E	OK	AL022_RS02415- *GyrB* 195bp<br>AL022_RS01165- *GroEL* 249bp<br>CAHE_0339 250 bp<br>CAHE_0131 191 bp<br>CAHE_0338 197 bp<br>AL022_RS01110 213 bp<br>CAHE_0242 174 bp<br>CAHE_0130 150 bp<br>CAHE_0475 188 bp<br>CAHE_0565 250 bp<br>CAHE_0102 225 bp<br>CAHE_p0027 182 bp<br>CAHE_0335 195 bp<br>CAHE_p0026 157 bp<br>CAHE_0132 171 bp<br>CAHE_0678 151 bp<br>CAHE_0544 233 bp
in silico specificity screen (blast, etc)	E	OK	done
Pseudogenes, retropseudogenes or other homologs?	D		
sequence alignment	D		
secondary structure analysis of amplicon	D	OK	
location of each primer by exon or intron (if applicable)	E		not relevant
What splice variants are targeted?	E		not relevant
**qPCR oligonucleotides**			
primer sequences	E	OK	see Supplementaty Table

RTPrimerdb identification number	D	OK	not relevant, as all of them are unpublished newly designed primers
probe sequences	D	OK	not relevant, as no probes were used
location and identity of any modifications	E	OK	no modifications
manufacturer of oligonucleotides	D	OK	Microsynth
purification method	D	OK	desalted
**qPCR protocol**			
complete reaction conditions	E	OK	See main manuscript
reaction volume and amount of cDNA/DNA	E	OK	See main manuscript
primer, (probe), Mg++ and dNTP concentrations	E	OK	See main manuscript
polymerase identity and concentration	E	OK	See main manuscript
buffer/kit identity and manufacturer	E	OK	See main manuscript
exact chemical constitution of the buffer	D	OK	See manual Brilliant III Ultra-Fast SYBR Green qPCR Master Mix (Agilent, Vienna, Austria)
Additives (SYBR green I, DMSO, etc.)	E	OK	SYBR Green is contained in the mastermix; no further additives
manufacturer of plates/tubes and catalog number	D	OK	MicroAmp optical tube (0.2 µl; Applied Biosystems by life technologies)
complete thermocycling parameters	E	OK	See main manuscript
reaction setup (manual/robotic)	D	OK	manual
manufacturer of qpcr instrument	E	OK	Stratagene Mx3000P real-time PCR System (Agilent Technologies, Santa Clara, USA)
**qPCR validation**			
evidence of optimisation (from gradients)	D	OK	done
specificity (gel, sequence, melt, or digest)	E	OK	melting curve
for SYBR green, Cq of the NTC	E	OK	no amplification
standard curves with slope and y-intercept	E	OK	done
PCR efficiency calculated from slope	E	OK	GyrB 92.3%<br>GroEL 99.3%<br>CAHE_0339 98.2%<br>CAHE_0131 122%<br>CAHE_0338 102%<br>RI14_RS01100 99.2%<br>CAHE_0242 97.9%<br>CAHE_0130 92.6%<br>CAHE_0475 92%<br>CAHE_0565 89%<br>CAHE_0102 95.6%<br>CAHE_p0027 103.9%<br>CAHE_0335 107.4%<br>CAHE_p0026 94.5%<br>CAHE_0132 101%<br>CAHE_0678 102.3%<br>CAHE_0544 92.7%

confidence interval for PCR efficiency or standard error	D		-
R2 of standard curve	E	OK	Between 0.98 and 1 for all primer pairs
linear dynamic range	E	OK	3 log scales tested
Cq variation at lower limit	E	OK	not tested
confidence intervals throughout range	D		
evidence for limit of detection	E	OK	not tested
If multiplex, efficiency and LOD of each assay.	E	OK	not relevant, no multiplexing
**data analysis**			
qPCR analysis program (source, version)	E		Stratagene Mx3000P real-time PCR System (Agilent Technologies, Santa Clara, USA)
Cq method determination	E		Stratagene Mx3000P real-time PCR System settings (baseline subtracted curve fit, single threshold, automatically calculated). Threshold manually curated for maximum efficiency within linear range for each plate
outlier identification and disposition	E	OK	done
results of NTCs	E	OK	no amplificate
justification of number and choice of reference genes	E	OK	Assumption that *gyrB* and *groEL* are constantly expressed. *GroEL* is the best-conserved chaperone, single copy.
description of normalisation method	E	OK	relative quantification – copy number of reference genes per sample normalised against copy number of reference mRNAs within the same sample
number and concordance of biological replicates	D	OK	3 biological replicates
number and stage (RT or qPCR) of technical replicates	E	OK	2 technical replicates for all samples
repeatability (intra-assay variation)	E	OK	repeatable
reproducibility (inter-assay variation, %CV)	D	OK	not determined (strongly recommended for clinical/diagnostic applications, but not other assays)
power analysis	D	OK	
statistical methods for result significance	E	OK	done
software (source, version)	E	OK	R software
Cq or raw data submission using RDML	D		
E = essential,<br>D = recommended			
